# Optimization of the Pharmacokinetic Profile of [^99m^Tc]Tc-N_4_-Bombesin Derivatives by Modification of the Pharmacophoric Gln-Trp Sequence

**DOI:** 10.3390/ph15091133

**Published:** 2022-09-10

**Authors:** Thomas Günther, Matthias Konrad, León Stopper, Jan-Philip Kunert, Sebastian Fischer, Roswitha Beck, Angela Casini, Hans-Jürgen Wester

**Affiliations:** Department of Chemistry, Technical University of Munich, 85748 Garching, Germany

**Keywords:** GRPR, technetium-99m, pharmacophore-modified compounds, prostate cancer, breast cancer

## Abstract

Current radiolabeled gastrin-releasing peptide receptor (GRPR) ligands usually suffer from high accumulation in GRPR-positive organs (pancreas, stomach), limiting tumor-to-background contrast in the abdomen. In novel N_4_-bombesin derivatives this was addressed by substitutions at the Gln^7^-Trp^8^ site within the MJ9 peptide (*H*-Pip^5^-phe^6^-Gln^7^-Trp^8^-Ala^9^-Val^10^-Gly^11^-His^12^-Sta^13^-Leu^14^-NH_2_) either by homoserine (Hse^7^), *β*-(3-benzothienyl) alanine (Bta^8^) or *α*-methyl tryptophan (*α*-Me-Trp^8^), with the aim of optimizing pharmacokinetics. We prepared and characterized the peptide conjugates 6-carboxy-1,4,8,11-tetraazaundecane (N_4_)-asp-MJ9, N_4_-asp-[Bta^8^]MJ9, N_4_-[Hse^7^]MJ9 and N_4_-[α-Me-Trp^8^]MJ9, and evaluated these compounds in vitro (GRPR affinity via *IC*_50,inverse_; internalization; lipophilicity via log*D*_7.4_) and in vivo (biodistribution and *μ*SPECT/CT studies at 1 h post injection (p.i.) in PC-3 tumor-bearing CB17-SCID mice). ^99m^Tc-labeling resulted in radiochemical yields (RCYs) > 95%. All ^99m^Tc-labeled MJ9 analogues showed comparable or higher GRPR affinity than the external reference [^99m^Tc]Tc-Demobesin 4. Receptor-bound fractions were noticeably higher than that of the reference. Despite a slightly enhanced lipophilicity, all novel MJ9 derivatives revealed improved in vivo pharmacokinetics compared to the reference. The Bta^8^-modified ligand revealed the most favorable tumor-to-abdomen contrast at 1 h p.i. Substitutions at the Gln^7^-Trp^8^ site within GRPR ligands hold great potential to modify pharmacokinetics for improved imaging.

## 1. Introduction

The field of nuclear medicine continues to grow worldwide, as the importance of non-invasive imaging modalities that bring forth biochemical information on various malignancies is continuously increasing [1,2]. Due to increased life expectancy, the probability of developing cancer will also be higher in the future, which is why the need for personalized medicine will rise as well [3]. Despite growing interest in positron emission tomography (PET) imaging as a consequence of its enhanced sensitivity, single-photon emission computed tomography (SPECT) devices are still more common due to lower acquisition costs, which is especially attractive for smaller nuclear medicine centers, and its widespread application in cardiology [4]. Another advantage of SPECT is the good availability of the generator-produced radionuclide technetium-99m that is perfectly suited for the SPECT technology due to its unique physical properties (half-life of ~6 h, E*_γ_* ~ 142 keV). Not surprisingly, technetium-99m is still the most used radionuclide in nuclear medicine, amounting to up to 80% of all nuclear medicine procedures worldwide [2]. Amongst the various chelators that are available for radiolabeling peptides [5,6,7,8], we selected 6-carboxy-1,4,8,11-tetraazaundecane (N_4_) because it stabilizes ^99m^Tc-complexes that display high stability in vivo, has good hydrophilicity, and has already been applied in preclinical and clinical settings for a variety of target structures [9].

Usually, these chelators are attached to a biomolecule that specifically addresses a target overexpressed by certain malignancies. With regard to prostate cancer, the most often addressed target is the prostate-specific membrane antigen (PSMA), which is overexpressed in approximately 90% of prostate cancers in high density. An often-applied PSMA-targeted compound for SPECT imaging is [^99m^Tc]Tc-MIP-1404, which revealed high uptake in the tumor (lesions) and the kidneys preclinically and clinically [10,11]. Another promising target with regard to prostate cancer [12] is the gastrin-releasing peptide receptor (GRPR), which was shown to be overexpressed not only in prostate but also in breast cancer [13], as well as gastrointestinal stromal tumors [14], amongst others. The bombesin-based peptide, MJ9 (*H*-Pip^5^-phe^6^-Gln^7^-Trp^8^-Ala^9^-Val^10^-Gly^11^-His^12^-Sta^13^-Leu^14^-NH_2_), possesses high affinity to GRPR and further showed attractive pharmacokinetics in vivo, independent of the *N*-terminal conjugated radiometal-chelator moiety [15,16,17]. While most bombesin-based compounds generally display favorable biodistribution pattern in animals and humans, high off-target accumulation is usually observed at early time points (1 h post injection, p.i.) in the abdominal region, mainly in the pancreas [18,19,20,21,22]. In healthy individuals, GRPR expression is observed to a high degree in the pancreas and to a lesser degree in the stomach [23]. In general, GRPR antagonists are cleared more rapidly from the pancreas than from the tumor over time, which is why tumor-to-pancreas ratios at 4 or 24 h p.i. are noticeably higher than at 1 h p.i. [17,24,25]. Moreover, GRPR antagonists display a noticeably faster activity clearance from the pancreas than GRPR agonists. It was observed that the activity retention of radiolabeled GRPR agonists in the pancreas remains high within the first 24 h p.i. [24]. As imaging in hospitals generally takes place within 1–2 h p.i. due to logistic reasons, a rapid activity clearance from healthy organs and, thus, high tumor-to-background contrast at early time points is desired, which is also beneficial with regard to dosimetry.

So far, published approaches to optimize the pharmacokinetics of bombesin-like peptides mostly focus on modifications at the *C*- and/or *N*-terminus [17,19,22,26] or the co-administration of a peptidase inhibitor to increase metabolic stability, and thus, the bioavailability of the respective compounds [20,25,27,28]. By an α-methyl-L-Trp-for-L-Trp^8^ substitution within the pharmacophore, we were also able to enhance the in vivo stability of bombesin-derived ligands, which is especially beneficial for targeted radiotherapy [29]. However, in our group, we also observed an interesting phenomenon of improved tumor-to-pancreas ratios at early time points when addressing the Gln^7^-Trp^8^ site by a homoserine (Hse)-for-Gln^7^ or a *β*-(3-benzothienyl) alanine (Bta)-for-Trp^8^ substitution. Interestingly, we found a lower metabolic stability for the respective [^177^Lu]Lu-DOTA-conjugated MJ9 analogues [30]. We assumed that metabolism in the pancreas is more rapid than in the tumor, which is why at early time points the less stable GRPR-targeted peptides exhibited an accelerated clearance from the pancreas but not from the tumor, compared to more stable analogues.

**Figure 1:**
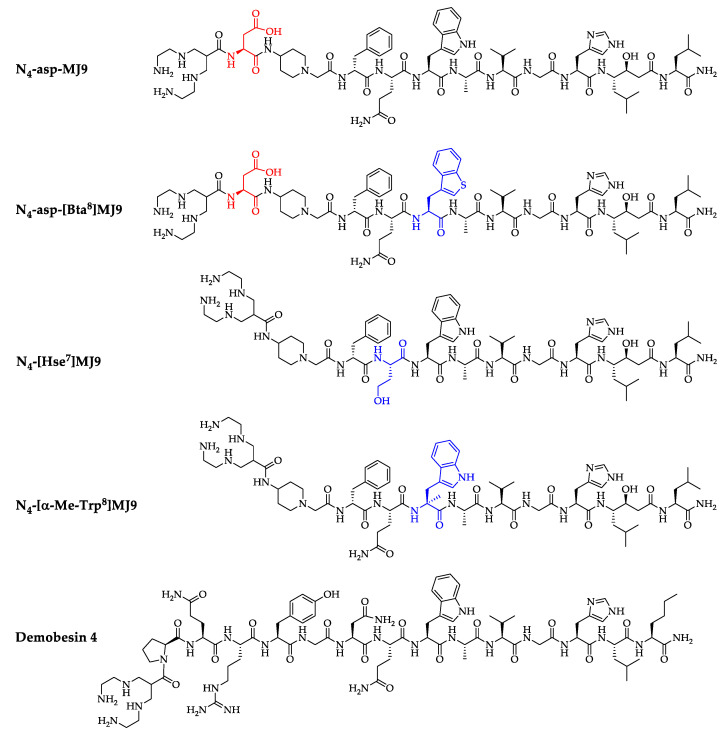
Chemical structures of N_4_-asp-MJ9, N_4_-asp-[Bta^8^]MJ9, N_4_-[Hse^7^]MJ9, N_4_-[α-Me-Trp^8^]MJ9 and the reference ligand Demobesin 4. Pharmacophoric modifications are highlighted in blue, while linker section modifications are highlighted in red.

As we aimed for improved imaging contrast in the abdominal region at early time points and reduced doses to the pancreas, we introduced Hse^7^ or Bta^8^ instead of Gln^7^ and Trp^8^ in MJ9, respectively. We thus wanted to investigate whether these substitutions show a similar positive influence on the early pharmacokinetics of [^99m^Tc]Tc-N_4_-conjugated MJ9 derivatives, as we recently observed for our [^177^Lu]Lu-DOTA-conjugated MJ9 analogues. Moreover, we also rendered an α-methyl-L-Trp-for-L-Trp^8^ substitution to determine the impact of this modification on early imaging, as it was originally intended to improve metabolic stability for targeted radiotherapy. We conjugated the N_4_ chelator and an additional D-aspartate moiety to reduce lipophilicity (for two out of four compounds) to the *N*-terminus and investigated the corresponding ^99m^Tc-labeled compounds alongside with the potent reference ligand [^99m^Tc]Tc-Demobesin 4 (Figure 1) in terms of affinity studies (*IC*_50,inverse_) on PC-3 cells, membrane-bound fractions determined via internalization studies on PC-3 cells, lipophilicity (log*D*_7.4_) and in vivo studies. We are aware that Demobesin 4 is a GRPR agonist and may thus show different pharmacokinetics than the four GRPR antagonists examined in this study. However, as [^99m^Tc]Tc-Demobesin 4 was already evaluated in a first-in-man study [31], we decided to include this compound as a reference, as we also aim to design GRPR ligands for future clinical application.

## 2. Results

### 2.1. Synthesis and Radiolabeling

The synthesis of unlabeled N_4_-conjugated GRPR-targeting peptides was carried out via standard Fmoc-based SPPS, which resulted in a 7–11% yield of each labeling precursor following purification by RP-HPLC (chemical purity > 94%, determined by RP-HPLC at *λ* = 220 nm, compound identity confirmed by ESI-MS, Figures S4a,c, S6a,c, S8a,c, S10a,c and S12a,c). The radiolabeling of all N_4_-conjugated compounds was carried out using freshly generator-eluted [^99m^Tc][TcO_4_]^−^, and each resulted in quantitative radiochemical yields (RCYs) and radiochemical purities (RCPs) > 95% (as determined by radio RP-HPLC, Figures S4b, S6b, S8b, S10b and S12b), as well as the molar activities of 33 ± 4 GBq/μmol. All ^99m^Tc-labeled compounds were used without further purification.

### 2.2. In Vitro Characterization

**Figure 2:**
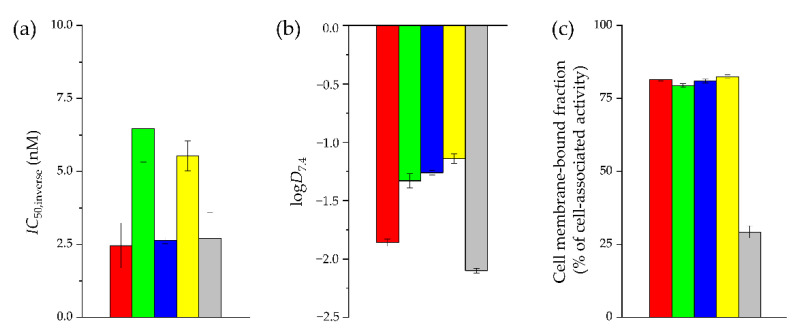
In vitro data of [^99m^Tc]Tc-N_4_-asp-MJ9 (red), [^99m^Tc]Tc-N_4_-asp-[Bta^8^]MJ9 (green), [^99m^Tc]Tc-N_4_-[Hse^7^]MJ9 (blue), [^99m^Tc]Tc-N_4_-[α-Me-Trp^8^]MJ9 (yellow) and [^99m^Tc]Tc-Demobesin 4 (grey): (**a**) Affinity of ^99m^Tc-labeled ligands (8.0 nM/well) on PC-3 cells (1.5 × 10^5^ cells/mL/well) using 3-I-tyr^6^-MJ9 (10^−10^–10^−4^ M) as non-radioactive competitor (2 h, room temperature, HBSS + 1% BSA, *v*/*v*); (**b**) Lipophilicity at pH 7.4 (log*D*_7.4_); (**c**) Cell membrane-bound fractions on PC-3 cells as percent of cell-associated activity, determined via internalization studies. Incubation at 37 °C for 1 h, DMEM/F-12 + 5% BSA (*v*/*v*), 1.5 × 10^5^ cells/mL/well, 0.25 pmol/well. Data corrected for non-specific binding (10^−3^ M [^nat^Lu]Lu-RM2).

In vitro data of all ^99m^Tc-labeled MJ9 derivatives as well as the external reference compound, [^99m^Tc]Tc-Demobesin 4, are summarized in Figure 2 and Table S1.

It has to be mentioned that in case of the inverse half maximal inhibitory concentration (*IC*_50,inverse_) studies, the respective radiolabeled MJ9 analogue is used at a defined concentration (8.0 nM/well), while the competitor applied (3-I-tyr^6^-MJ9) is used in non-radioactive and in rising concentrations (final concentrations of 10^−11^–10^−5^ M/well). Hence, in contrast to conventional *IC*_50_ values, higher *IC*_50,inverse_ values refer to higher (GRPR) affinity, as a higher concentration of 3-I-tyr^6^-MJ9 is required to displace the respective radiolabeled MJ9 derivative from the receptor.

The radiolabeled counterpart (3-[^125^I]I-tyr^6^-MJ9) of the non-radioactive competitor (3-I-tyr^6^-MJ9, conventional *IC*_50_ of 1.3 ± 0.4 nM) showed an *IC*_50,inverse_ of 4.9 ± 0.9 nM on PC-3 cells and was considered a valid competitor in our cell-based assay. While both [^99m^Tc]Tc-N_4_-asp-MJ9 and [^99m^Tc]Tc-N_4_-[Hse^7^]MJ9 revealed *IC*_50,inverse_ values similar to the reference [^99m^Tc]Tc-Demobesin 4 (2.5–2.7 nM, *p* > 0.38), the two ligands modified at the Trp^8^ position, [^99m^Tc]Tc-N_4_-asp-[Bta^8^]MJ9 and [^99m^Tc]Tc-N_4_-[α-Me-Trp^8^]MJ9 (5.5–6.5 nM, *p* > 0.16), exhibited significantly increased *IC*_50,inverse_ values on PC-3 cells, and thus, enhanced GRPR affinity (*p* < 0.02). All ^99m^Tc-labeled MJ9 analogues demonstrated noticeably or slightly increased lipophilicity (log*D*_7.4_ values of −1.8 to −1.1) compared to [^99m^Tc]Tc-Demobesin 4 (−2.10 ± 0.02), which was statistically significant (*p* < 0.0001). Furthermore, the MJ9 derivatives revealed high cell membrane-bound fractions on PC-3 cells (1.5 × 10^5^ cells/well) within 1 h (79–83% of cell-associated activity, *p* > 0.46), while internalized fractions were low (17–21% of cell-associated activity). Not surprisingly, a low membrane-bound fraction (~29% of cell-associated activity) was found for the GRPR agonist [^99m^Tc]Tc-Demobesin 4, while internalized fractions were high (~71% of cell-associated activity), which was statistically significant compared to the GRPR antagonists (*p* < 0.03).

### 2.3. In Vivo Characterization

**Figure 3:**
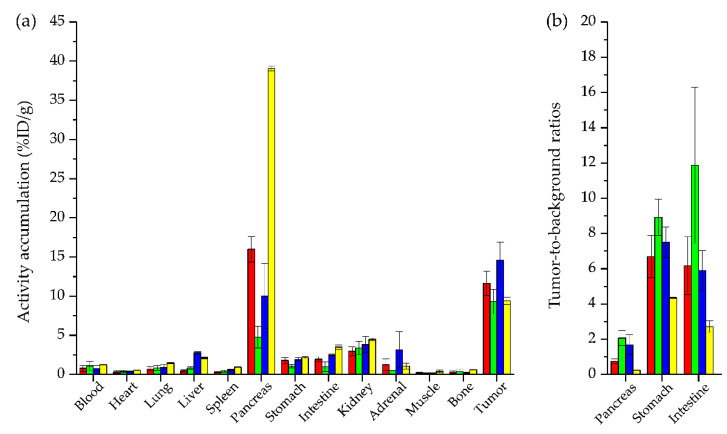
Biodistribution data of [^99m^Tc]Tc-N_4_-asp-MJ9 (red), [^99m^Tc]Tc-N_4_-asp-[Bta^8^]MJ9 (green), [^99m^Tc]Tc-N_4_-[Hse^7^]MJ9 (blue) and [^99m^Tc]Tc-N_4_-[α-Me-Trp^8^]MJ9 (yellow). (**a**) Biodistribution of the ^99m^Tc-labeled MJ9 derivatives (200 pmol each) in selected organs at 1 h p.i. in PC-3 tumor-bearing CB17-SCID mice. Data are %ID/g, mean ± SD (*n* = 3). (**b**) Tumor-to-background ratios of the ^99m^Tc-labeled MJ9 derivatives for selected organs at 1 h p.i. in PC-3 tumor-bearing CB17-SCID mice. Data are mean ± SD (*n* = 3).

Biodistribution studies in PC-3 tumor-bearing mice were carried out at 1 h post injection (p.i.) (Table 1, Figure 3).

All ^99m^Tc-labeled MJ9 derivatives revealed similarly low activity levels in all organs (<4.5 percent injected dose per gram; %ID/g), except for the GRPR-positive pancreas (4.7–39.0 %ID/g) and the PC-3 tumor (9.3–14.6 %ID/g) at 1 h p.i., which indicates a rapid clearance from off-target tissues. It is worth noting that both [^99m^Tc]Tc-N_4_-asp-[Bta^8^]MJ9 and [^99m^Tc]Tc-N_4_-[Hse^7^]MJ9 exhibited less activity levels in the pancreas than [^99m^Tc]Tc-N_4_-asp-MJ9 (*p* < 0.03), which was not modified within the pharmacophore. While [^99m^Tc]Tc-N_4_-asp-[Bta^8^]MJ9 showed slightly reduced levels, [^99m^Tc]Tc-N_4_-[Hse^7^]MJ9 displayed slightly enhanced activity levels in the tumor compared to [^99m^Tc]Tc-N_4_-asp-MJ9, which were not statistically significant (*p* > 0.05). Interestingly, [^99m^Tc]Tc-N_4_-[α-Me-Trp^8^]MJ9 demonstrated similarly high uptake values in the tumor and the pancreas to [^99m^Tc]Tc-Demobesin 4 (values determined by Nock et al. [32]) at 1 h p.i. (Table 1) but lower activity levels in the kidneys and intestine. In general, these two compounds revealed the highest off-target accumulation within this series. Not surprisingly, [^99m^Tc]Tc-N_4_-asp-[Bta^8^]MJ9 (*p* < 0.02) and [^99m^Tc]Tc-N_4_-[Hse^7^]MJ9 (*p* < 0.01) showed the highest tumor-to-pancreas ratios at 1 h p.i. Nevertheless, it has to be mentioned that a comparison of in vivo data for [^99m^Tc]Tc-Demobesin 4 and the four novel [^99m^Tc]Tc-N_4_-conjugated MJ9 analogues must be drawn cautiously, as besides the different behavior of GRPR agonists and antagonists, different amounts of substance and animal strains were applied.

**Figure 4:**
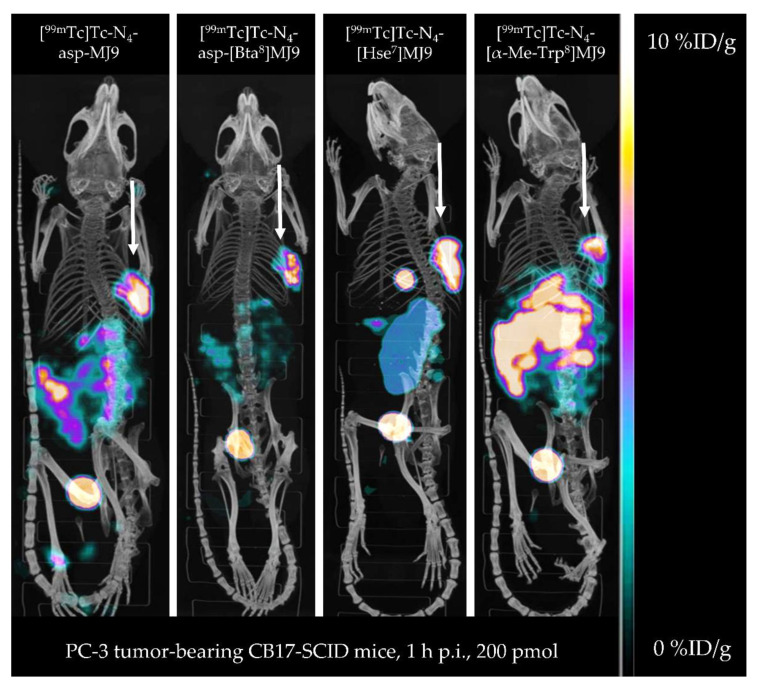
Maximum-intensity projection of PC-3 tumor-bearing CB17-SCID mice injected with [^99m^Tc]Tc-N_4_-asp-MJ9, [^99m^Tc]Tc-N_4_-asp-[Bta^8^]MJ9, [^99m^Tc]Tc-N_4_-[Hse^7^]MJ9 and [^99m^Tc]Tc-N_4_-[α-Me-Trp^8^]MJ9 (200 pmol each, *n* = 1). Images were acquired at 1 h p.i. PC-3 tumors are depicted by white arrows.

*µ*SPECT/CT studies with the ^99m^Tc-labeled MJ9 derivatives at 1 h p.i. in PC-3 tumor-bearing mice (Figure 4) confirmed the biodistribution data, with [^99m^Tc]Tc-N_4_-asp-[Bta^8^]MJ9 displaying the highest tumor-to-abdomen contrast at 1 h p.i. due to a rapid clearance from GRPR-positive pancreas and stomach. Furthermore, high activity levels in the tumor were observed for both [^99m^Tc]Tc-N_4_-asp-MJ9 and [^99m^Tc]Tc-N_4_-[Hse^7^]MJ9. However, the latter demonstrated lower activity levels in the abdominal region, except for the gall bladder, which showed unexpectedly high levels. As expected, [^99m^Tc]Tc-N_4_-[α-Me-Trp^8^]MJ9 revealed a low tumor-to-abdomen contrast at 1 h p.i. due to its high pancreatic uptake.

## 3. Discussion

Due to its unique physical properties and widespread availability, the cost-effective generator-produced radionuclide technetium-99m had and still has a great impact on nuclear medicine. To date, the use of ^99m^Tc-labeled compounds for single-photon emission computed tomography (SPECT) or SPECT/computed tomography (SPECT/CT) is highly established and used most commonly [2]. For this reason, we decided to design a series of pharmacophore-modified GRPR ligands that allow for easy and rapid ^99m^Tc-labeling via a 6-carboxy-1,4,8,11-tetraazaundecane (N_4_) chelator conjugated to the *N*-terminus, and which show improved tumor-to-abdomen contrast, as bombesin-derived compounds tend to accumulate mainly in the pancreas, but also in the stomach (both GRPR-positive organs) and the intestine [17,22,32,33]. Prior work of our group on ^177^Lu-labeled GRPR antagonists with modifications at the Gln^7^-Trp^8^ site within the pharmacophore [29] in the biological behavior of bombesin derivatives revealed that the introduction of homoserine (Hse^7^) and *β*-(3-benzothienyl) alanine (Bta^8^) into the MJ9 (*H*-Pip^5^-phe^6^-Gln^7^-Trp^8^-Ala^9^-Val^10^-Gly^11^-His^12^-Sta^13^-Leu^14^-NH_2_) peptide might result in increased tumor-to-abdomen ratios. We therefore transferred this concept to [^99m^Tc]Tc-N_4_-conjugated MJ9 derivatives and compared the resulting ligands to the unmodified, as well as the α-methyl-tryptophan (α-Me-Trp^8^)-containing analogue in vitro and in vivo.

**Figure 5:**
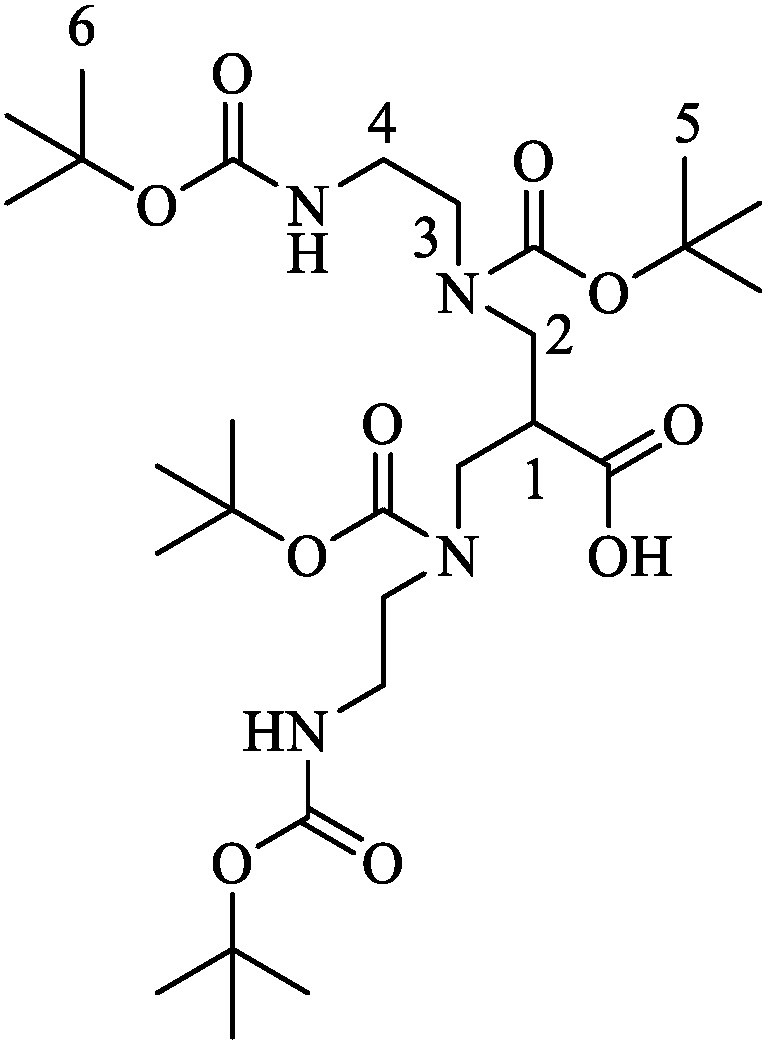
Characterization of (*^t^*Bu)_4_N_4_ by ^1^H-NMR (400 MHz, DMSO-*d*_6_) *δ* = 6.79 (s, 2H, NH), 3.28–3.17 (m, 4H, C4-H_2_), 3.22–3.04 (m, 4H, C3-H_2_), 3.10–2.95 (m, 4H, C2-H_2_), 2.94–2.90 (m, 1H, C1-H), 1.38 (s, 18H, C5-H_3_), 1.36 (s, 18H, C6-H_3_).

Identity of (*^t^*Bu)_4_N_4_ was confirmed by ^1^H-NMR (Figure 5, see chapter 4.1). Synthesis via solid-phase peptide synthesis and ^99m^Tc-labeling of the respective peptides was easily accessible and resulted in RCYs > 95% and molar activities of 33 ± 4 GBq/μmol. In later experiments, we were able to achieve molar activities of up to 60 GBq/µmol by reducing the total volume and adding sodium ascorbate only after labeling. Abiraj et al. reported on a molar activity of up to 100 GBq/µmol for somatostatin analogues, while Nock et al. reached molar activities of 18–37 and >50 GBq/µmol for Demobesin analogues labeled by a similar protocol [18,22,34].

All ^99m^Tc-labeled MJ9 derivatives revealed high GRPR affinity (*IC*_50,inverse_: 2.4–6.3 nM). We decided to determine inverse *IC*_50_ values, as we wanted to investigate the GRPR affinity of the ^99m^Tc-labeled compounds rather than the unlabeled precursors. We are convinced that there could be an influence of an unlabeled compared to a labeled chelator moiety, which is why in the case of technetium-99m we prefer inverse *IC*_50_ values. Most groups only determine conventional *IC*_50_ values of the respective unlabeled precursors, a comparison to our *IC*_50,inverse_ values is difficult [18,22,32]. Abouzayed et al. reported on equilibrium dissociation constants in the (low) picomolar range, which is also difficult to compare with our *IC*_50,inverse_ values [35]. Therefore, we included the well-known GRPR ligand [^99m^Tc]Tc-Demobesin 4 as an external reference compound, which exhibited an *IC*_50,inverse_ value of approximately 2.5 nM. Moreover, the non-radioactive competitor (3-I-tyr^6^-MJ9), which revealed an *IC*_50_ value of 1.3 ± 0.4 nM in previous studies [29], showed an *IC*_50,inverse_ value of 4.9 ± 0.9 nM for its ^125^I-labeled counterpart in this study. We thus concluded that each compound comprised sufficiently high GRPR affinity for further studies in vivo.

Furthermore, we also suggested antagonistic behavior for all novel MJ9 analogues due to their high structural similarity to the well-known GRPR antagonist ([^68^Ga]Ga-/[^177^Lu]Lu-) RM2, as well as their low internalization percentage determined in the present study. In contrast, it was reported that the GRPR agonist [^99m^Tc]Tc-Demobesin 4 displays a GRPR-mediated internalization of ~75% on PC-3 cells [32], which corroborates with our data.

The substitution of a [^177^Lu]Lu-DOTA by a [^99m^Tc]Tc-N_4_ moiety led to a drastic increase in lipophilicity, as previously developed compounds exhibited log*D*_7.4_ values of ≤−1.8 [36], while the ^99m^Tc-labeled MJ9 derivatives revealed values in a range of −1.8 to −1.1. By the introduction of a D-aspartate moiety, lipophilicity could be slightly reduced, but it remained elevated compared to [^177^Lu]Lu-DOTA-conjugated derivatives. However, we considered this lipophilicity sufficient for in vivo studies, as the clinically applied GRPR ligand [^68^Ga]Ga-/[^177^Lu]Lu-NeoBOMB1 demonstrated log*D*_7.4_ values of −0.9 and −0.6, respectively [29,37]. We are aware that the introduction of a D-aspartate moiety into the *N*-terminal linker section could also affect other in vivo parameters, such as cell uptake and metabolic stability, amongst others, as it was observed for a GRPR antagonist that even the change of the radiometal can significantly alter these parameters [38]. Therefore, comparisons between [^99m^Tc]Tc-N_4_-asp-MJ9 or [^99m^Tc]Tc-N_4_-asp-[Bta^8^]MJ9, both containing said D-aspartate moiety, and [^99m^Tc]Tc-N_4_-[Hse^7^]MJ9 or [^99m^Tc]Tc-N_4_-[α-Me-Trp^8^]MJ9, both not comprising a D-aspartate moiety, have to be rendered cautiously.

Despite slightly increased liver uptake observed for [^99m^Tc]Tc-N_4_-[Hse^7^]MJ9 and [^99m^Tc]Tc-N_4_-[α-Me-Trp^8^]MJ9 at 1 h p.i., activity levels in further GRPR-negative organs were generally low. Therefore, none of the ^99m^Tc-labeled MJ9 derivatives showed significant limitations in vivo as a consequence of enhanced lipophilicity. Interestingly, [^99m^Tc]Tc-N_4_-asp-[Bta^8^]MJ9, which also displayed increased lipophilicity, did not show enhanced activity levels in the liver. Moreover, activity levels in GRPR-positive organs were noticeably reduced (pancreas by 70% and stomach by 40%) while tumor uptake was only decreased by 20% compared to [^99m^Tc]Tc-N_4_-asp-MJ9 at 1 h p.i., pointing to a beneficial impact of the Bta-for-Trp^8^ substitution on clearance kinetics.

Surprisingly, [^99m^Tc]Tc-N_4_-[Hse^7^]MJ9 revealed the highest tumor uptake of all compounds at 1 h p.i., although it did not show the highest GRPR affinity, while [^99m^Tc]Tc-N_4_-[α-Me-Trp^8^]MJ9, which revealed the second highest GRPR affinity, exhibited the second lowest activity levels in the tumor at 1 h p.i. Moreover, the latter conjugate displayed enhanced activity levels in most non-tumor organs, especially visible in the pancreas. For this reason, the α-Me-Trp-for-Trp^8^ substitution within the MJ9 sequence did not lead to improved imaging properties.

A limitation of this study is the absence of competition studies to confirm the tumor (and pancreas) specificity of the novel [^99m^Tc]Tc-labeled MJ9 derivatives. Previous studies revealed GRPR-specific tumor (and pancreas) accumulation for the respective [^177^Lu]Lu-DOTA-conjugated MJ9 analogues [29,30], as other MJ9 derivatives described in the literature also displayed a GRPR-specific uptake pattern and GRPR affinity was high for all novel [^99m^Tc]Tc-labeled MJ9 derivatives. Moreover, internalization studies revealed that >99% of the applied activity could be blocked by an excess of [^nat^Lu]Lu-RM2. For all these reasons, we suggest that there is a high probability that the tumor and pancreas uptake of the novel [^99m^Tc]Tc-N_4_-conjugated MJ9 analogues is GRPR-specific. Nevertheless, this has to be addressed in future studies to confirm.

A study on [^99m^Tc]Tc-Demobesin 4 showed activity levels of ~11 %ID/g in the tumor at 1 h p.i., but also distinctly higher levels in the pancreas (~40 %ID/g), the intestine (~8 %ID/g) and the kidneys (~20 %ID/g), compared to our MJ9 derivatives [32]. Similar values were reported for [^99m^Tc]Tc-N_4_-GRP_14–27_ without the addition of a neprilysin inhibitor [39]. However, a comparison of the in vivo data of GRPR agonists and antagonists as well as the effect of using different amounts of substance is difficult, as it was observed that clearance kinetics might be affected [24,33]. Moreover, different animal strains were applied, which can also have an influence on overall pharmacokinetics [40]. Further studies on ^99m^Tc-labeled Demobesin derivatives revealed activity levels in the tumor of up to 30 %ID/g but also noticeably enhanced levels in the pancreas (up to 180 %ID/g) [18,22]. Makris et al. reported on NOTA- and NODAGA-conjugated MJ9 derivatives that exhibited activity levels of ~8 %ID/g in the tumor, but also enhanced levels in the pancreas and the intestine (~14 %ID/g each) at 1 h p.i. [41].

While all these compounds suffered from increased activity levels in the pancreas, we successfully addressed this issue by substituting the Gln^7^-Trp^8^ bond within the pharmacophore of MJ9 analogues either by Hse^7^ or Bta^8^ to improve tumor-to-abdomen contrast, which is attributed to a more rapid activity clearance from healthy organs. Interestingly, Wang et al. recently reported on a series of bombesin analogues modified at the *C*-terminus, which resulted in high activity levels in the tumor (~16 %ID/g) and low levels in the pancreas (~2 %ID/g) at 1 h p.i. for one compound ([^68^Ga]Ga-TacsBOMB5) [26]. The authors suggested that [^68^Ga]Ga-TacsBOMB5 might be more selective for the human than for the murine GRPR. Such interspecies differences between human and animal GRPR were also observed by Maina et al. [42].

Compared to ^99m^Tc-labeled PSMA inhibitors such as [^99m^Tc]Tc-MIP-1404, our MJ9 analogues revealed comparable or even higher activity levels in the tumor and noticeably lower levels in the kidneys [10] in tumor-bearing animals at 1 h p.i., which was expected, as the latter organ is PSMA-positive but GRPR-negative. However, in general, off-target accumulation was lower for the PSMA inhibitor than for the GRPR-targeted compounds at 1 h p.i., with the exception of the kidneys. We are aware that the comparison of different tumor models and target structures is difficult. Nevertheless, as particularly [^99m^Tc]Tc-N_4_-asp-[Bta^8^]MJ9 revealed a favorable biodistribution profile and [^99m^Tc]Tc-MIP-1404 demonstrated high uptake in liver and duodenum in prostate cancer patients, there might be a potential for the GRPR ligand that exhibited improved tumor-to-abdomen contrast preclinically.

In summary, we successfully designed pharmacophore-modified MJ9 analogues for ^99m^Tc-labeling with comparable or even higher GRPR affinity, low internalization but increased lipophilicity. While biodistribution profiles did not confirm our concerns regarding increased lipophilicity, future compounds with a decreased lipophilicity will show whether the contrast in the abdominal area that was already improved in this study can be optimized even further. We could confirm our hypothesis of improving tumor-to-non-tumor organ ratios in the abdomen at early time points by modifying the pharmacophore of the MJ9 peptide either by Hse^7^ or Bta^8^. Due to recent observations in our group for [^177^Lu]Lu-conjugated MJ9 analogues with regard to in vivo stability, future studies have to elucidate whether this beneficial clearance effect might also be linked to a decreased metabolic stability of the novel [^99m^Tc]Tc-N_4_-conjugated MJ9 derivatives. Nevertheless, based on the preclinical data determined in this work, [^99m^Tc]Tc-N_4_-asp-[Bta^8^]MJ9 might be a potential candidate for clinical translation because of its favorable contrast at early imaging time points. In general, bombesin-based tracers could take a complementary role to PSMA inhibitors for prostate cancers and, furthermore, might also be applicable for the imaging of breast cancer and gastrointestinal stromal tumors. It seems plausible to conclude that other GRPR ligands sharing the same pharmacophore (Gln^7^-Trp^8^-Ala^9^-Val^10^-Gly^11^-His^12^) might also benefit from substitutions at the Gln^7^-Trp^8^ site.

## 4. Materials and Methods

Further general information and characterization of the non-radioactive competitor (3-I-tyr^6^-MJ9), its radiolabeled analogue (3-[^125^I]I-tyr^6^-MJ9), all N_4_-conjugated MJ9 analogues and the external reference is provided in the supplemental Material (Figures S1–S12). Electrospray ionization-mass spectra for characterization of the substances were acquired on an expression^L^ CMS mass spectrometer (Advion Ltd., Harlow, UK). ^1^H-NMR-spectra were acquired on an AVHD 400 from Bruker (Billerica, MA, USA) at 300 K. Chemical shifts (*δ*) are given in parts per million (ppm), spectra are calibrated to the residual ^1^H solvent signal of DMSO-*d*_6_ at 2.50 ppm and signal multiplicities are described as: s = singlet, m = multiplet.

### 4.1. Synthesis of N,N′,N″,N‴-Tetrakis(tert-butyloxycarbonyl)-6-carboxy-1,4,8,11-tetraazaundecane ((^t^Bu)_4_N_4_)

*N*-Boc-ethylenediamine (4.0 eq.) was slowly added to a solution of 3-bromo-2-(bromomethyl)propionic acid (1.0 eq.) in THF (25 mL/mmol) and stirred for at least 24 h at room temperature. The solvent was removed under reduced pressure at room temperature and the residue was dissolved in acetone/H_2_O (1/1 (*v*/*v*), 25 mL/mmol). After the solution was cooled to 0 °C, triethylamine (3.0 eq.) was added. After 5 min, di-*tert*-butyl dicarbonate (4.0 eq.) was added and the mixture was stirred for 15 h (0 °C→room temperature). Subsequently, the solvents were removed under reduced pressure and the raw product was purified via reversed phase high performance flash chromatography (35–71% MeCN in H_2_O in 15 min), which yielded the desired product as a colorless solid.

ESI-MS: Calculated monoisotopic mass (C_28_H_52_N_4_O_10_): 604.4; found: *m*/*z* = 605.5 [M + H]^+^.

### 4.2. Compound Synthesis and Labeling Procedures

MJ9 derivatives and the non-radioactive competitor 3-I-tyr^6^-MJ9 were prepared via standard Fmoc-based solid phase peptide synthesis (SPPS) using a *H*-Rink amide ChemMatrix^®^ resin (35–100 mesh particle size, 0.4–0.6 mmol/g loading, Merck KGaA, Darmstadt, Germany). Purification was accomplished by semi-preparative reversed phase high performance liquid chromatography (RP-HPLC). Compound identity was confirmed via electrospray ionization mass spectrometry (ESI-MS) that was acquired on an expression^L^ CMS mass spectrometer (Advion Ltd., Harlow, UK).

^99m^Tc-Labeling of N_4_-conjugated GRPR ligands was carried out by the addition of 1.0 nmol precursor (10^−3^ M in Tracepur^®^ water) to a mixture of 25 µL of Na_2_HPO_4_ (0.05 M in Tracepur^®^ water, pH = 9.5) and 3.0 µL of disodium citrate sesquihydrate (0.1 M in Tracepur^®^ water) in saline. After the addition of a freshly prepared solution of 5.0 µL of SnCl_2_ (1 mg/mL ethanol), approximately 40 MBq [^99m^Tc][TcO_4_]^−^ in saline was added and the labeling solution (final volume of 150–750 µL) was heated to 90 °C for 10 min. Quality control was performed using radio thin layer chromatography and radio RP-HPLC (radio detection and UV detection at λ = 220 nm).

### 4.3. In Vitro Experiments

*Lipophilicity (logD_7.4_) and internalization*. The determination of both lipophilicity and cell membrane-bound as well as internalized fractions (PC-3 cells, 1.5 × 10^5^ cells/mL/well) was carried out with ^99m^Tc-labeled compounds in analogy to a published procedure [29].

*Affinity Determinations (IC_50,inverse_).* Competitive binding studies were performed in analogy to a previously reported procedure but with slight modifications [29]. The non-radioactive competitor (3-I-tyr^6^-MJ9) was applied in increasing concentrations (final concentration of 10^−11^–10^−5^ M/well), while novel ^99m^Tc-labeled GRPR ligands were used as radioligand (final concentration of 8.0 nM/well). All experiments were performed in triplicate for each concentration. *IC*_50,inverse_ determination for each compound was repeated twice. Data are mean ± standard deviation (SD).

### 4.4. In Vivo Experiments

All animal experiments were conducted in accordance with general animal welfare regulations in Germany (German animal protection act, in the edition of the announcement, dated 18 May 2006, as amended by Article 280 of 19 June 2020, approval no. ROB-55.2-1-2532.Vet_02-18-109 by the General Administration of Upper Bavaria) and the institutional guidelines for the care and use of animals. CB17-SCID mice of both genders and aged 2–10 months (Charles River Laboratories International Inc., Sulzfeld, Germany) were allowed to acclimate at the in-house animal facility for at least one week before inoculation was performed. The establishment of PC-3 tumor xenografts was carried out as previously published [29]. Exclusion criteria for animals from an experiment were either weight loss higher than 20%, a tumor size above 1500 mm^3^, an ulceration of the tumor, respiratory distress or a change of behavior. None of these criteria applied to any animal from the experiment. Neither randomization nor blinding was applied in the allocation of the experiments. Health status is SPF according to FELASA.

*Biodistribution and µSPECT/CT Imaging Studies.* Biodistribution (*n* = 3) and imaging (*n* = 1) studies were performed according to an established protocol using approximately 2–4 MBq (200 pmol, 150 µL) of the ^99m^Tc-labeled GRPR-targeted compounds [29]. The animals were euthanized at 1 h p.i. Acquired data were statistically analyzed by performing a Student’s *t*-test via Excel (Microsoft Corporation, Redmond, WA, USA) and OriginPro software (version 9.7) from OriginLab Corporation (Northampton, MA, USA). Acquired *p*-values of <0.05 were considered statistically significant.

Imaging studies were carried out according to a recently published protocol [29]. Static images were recorded at t = 1 (anesthesia by 2% isoflurane, *n* = 1) with an acquisition time of t + (45–60 min) using a high-energy general-purpose rat and mouse collimator via *MILabs* acquisition software v11.00 and v12.26 from MILabs (Utrecht, the Netherlands).

## 5. Conclusions

We demonstrated that our concept of modifying the pharmacophore of ^99m^Tc-labeled GRPR antagonists at the Gln^7^-Trp^8^ results in improved tumor-to-organ ratios in the abdominal area at early time points. In addition, we expect that addressing this dipeptide sequence in bombesin analogues might be a valuable tool to modify pharmacokinetics for both imaging and radioligand therapy.

## 6. Patents

A patent application on modified GRPR-targeted ligands with TG and HJW as inventors has been filed (WO2021121734A1).

## Figures and Tables

**Table 1:** Biodistribution data of [^99m^Tc]Tc-N_4_-asp-MJ9, [^99m^Tc]Tc-N_4_-asp-[Bta^8^]MJ9, [^99m^Tc]Tc-N_4_-[Hse^7^]MJ9 and [^99m^Tc]Tc-N_4_-[α-Me-Trp^8^]MJ9 (200 pmol each) compared to [^99m^Tc]Tc-Demobesin 4 at 1 h p.i. in PC-3 tumor-bearing CB17-SCID mice. * 10 pmol, 2–3 months old female Swiss nu/nu mice, data determined by Nock et al. [32].

Organs	[^99m^Tc]Tc-N_4_-asp-MJ9	[^99m^Tc]Tc-N_4_-asp-[Bta^8^]MJ9	[^99m^Tc]Tc-N_4_-[Hse^7^]MJ9	[^99m^Tc]Tc-N_4_-[α-Me-Trp^8^]MJ9	[^99m^Tc]Tc-Demobesin 4 *
Blood	0.79 ± 0.39	1.11 ± 0.55	0.77 ± 0.34	1.24 ± 0.01	~1.3 ± 0.5
Heart	0.32 ± 0.18	0.40 ± 0.16	0.36 ± 0.10	0.54 ± 0.04	n.a.
Lung	0.70 ± 0.27	0.83 ± 0.30	0.88 ± 0.39	1.46 ± 0.07	n.a.
Liver	0.48 ± 0.14	0.84 ± 0.16	2.78 ± 0.16	2.11 ± 0.13	~2.5 ± 0.3
Spleen	0.31 ± 0.10	0.43 ± 0.13	0.59 ± 0.18	0.95 ± 0.02	n.a.
Pancreas	15.99 ± 1.62	4.77 ± 1.38	10.00 ± 4.17	39.06 ± 0.28	~39.0 ± 7.0
Stomach	1.79 ± 0.38	1.07 ± 0.25	1.94 ± 0.19	2.17 ± 0.13	~2.4 ± 0.4
Intestine	1.96 ± 0.31	1.00 ± 0.61	2.49 ± 0.13	3.50 ± 0.26	~7.5 ± 1.5
Kidney	2.98 ± 0.57	3.37 ± 0.87	3.84 ± 1.00	4.49 ± 0.17	20.0 ± 4.0
Adrenal	1.25 ± 0.74	0.53 ± 0.04	3.17 ± 2.31	1.07 ± 0.36	n.a.
Muscle	0.17 ± 0.09	0.19 ± 0.05	0.16 ± 0.04	0.40 ± 0.17	~0.25 ± 0.05
Bone	0.30 ± 0.14	0.37 ± 0.28	0.22 ± 0.08	0.61 ± 0.05	n.a.
Tumor	11.66 ± 1.55	9.31 ± 1.56	14.59 ± 2.28	9.42 ± 0.46	~10.5 ± 0.5

Data are mean %ID/g ± SD (*n* = 3).

## Data Availability

Data is contained within the article and Supplementary Material.

## Supplementary Materials

The following supporting information can be downloaded at: https://www.mdpi.com/article/10.3390/ph15091133/s1, general information and characterization of all N_4_-conjugated MJ9 analogues, detailed description of cell-based experiments and animal studies. Figure S1: Structural formula of the radiolabeled counterpart (3-[^125^I]I-tyr^6^-MJ9) of the non-radioactive competitor (3-I-tyr^6^-MJ9) in *IC*_50_ studies; Figure S2: (a) Confirmation of peptide integrity for 3-[^125^I]I-tyr^6^-MJ9 (black), as analyzed by analytical (radio) RP-HPLC via co-injection of the non-radioactive ligand (blue). (b) Mass spectrum of 3-I-tyr^6^-MJ9; Figure S3: Structural formula of N_4_-asp-MJ9; Figure S4: (a) Confirmation of peptide identity and integrity for N_4_-asp-MJ9, as analyzed by analytical RP-HPLC. (b) Confirmation of peptide identity and integrity for [^99m^Tc]Tc-N_4_-asp-MJ9, as analyzed by analytical (radio) RP-HPLC. (c) Mass spectrum of N_4_-asp-MJ9; Figure S5: Structural formula of N_4_-asp-[Bta^8^]MJ9; Figure S6: (a) Confirmation of peptide identity and integrity for N_4_-asp-[Bta^8^]MJ9, as analyzed by analytical RP-HPLC. (b) Confirmation of peptide identity and integrity for [^99m^Tc]Tc-N_4_-asp-[Bta^8^]MJ9, as analyzed by analytical (radio) RP-HPLC. (c) Mass spectrum of N_4_-asp-[Bta^8^]MJ9; Figure S7: Structural formula of N_4_-[Hse^7^]MJ9; Figure S8: (a) Confirmation of peptide identity and integrity for N_4_-[Hse^7^]MJ9, as analyzed by analytical RP-HPLC. (b) Confirmation of peptide identity and integrity for [^99m^Tc]Tc-N_4_-[Hse^7^]MJ9, as analyzed by analytical (radio) RP-HPLC. (c) Mass spectrum of N_4_-[Hse^7^]MJ9; Figure S9: Structural formula of N_4_-[α-Me-Trp^8^]MJ9; Figure S10: (a) Confirmation of peptide identity and integrity for N_4_-[α-Me-Trp^8^]MJ9, as analyzed by analytical RP-HPLC. (b) Confirmation of peptide identity and integrity for [^99m^Tc]Tc-N_4_-[α-Me-Trp^8^]MJ9, as analyzed by analytical (radio) RP-HPLC. (c) Mass spectrum of N_4_-[α-Me-Trp^8^]MJ9; Figure S11: Structural formula of the external reference Demobesin 4; Figure S12: (a) Confirmation of peptide identity and integrity for Demobesin 4, as analyzed by analytical RP-HPLC. (b) Confirmation of peptide identity and integrity for [^99m^Tc]Tc-Demobesin 4, as analyzed by analytical (radio) RP-HPLC. (c) Mass spectrum of Demobesin 4; Table S1: Preclinical data of [^99m^Tc]Tc-N_4_-asp-MJ9, [^99m^Tc]Tc-N_4_-asp-[Bta^8^]MJ9, [^99m^Tc]Tc-N_4_-[Hse^7^]MJ9, [^99m^Tc]Tc-N_4_-[α-Me-Trp^8^]MJ9 and [^99m^Tc]Tc-Demobesin-4. Affinity data of the respective ^99m^Tc-labeled ligand (8.0 nM/well) was determined on PC-3 cells (1.5 × 10^5^ cells/well) and 3-I-tyr^6^-MJ9 (10^−10^–10^−4^ M) as non-radioactive competitor (2 h, room temperature, HBSS + 1% BSA, *v*/*v*). Lipophilicity was examined as distribution coefficient in a mixture of n-octanol/PBS (*v*/*v* = 1/1) at pH 7.4 (log*D*_7.4_). Cell membrane-bound fraction of [^99m^Tc]Tc-N_4_-asp-MJ9, [^99m^Tc]Tc-N_4_-asp-[Bta^8^]MJ9, [^99m^Tc]Tc-N_4_-[Hse^7^]MJ9, [^99m^Tc]Tc-N_4_-[α-Me-Trp^8^]MJ9 and [^99m^Tc]Tc-Demobesin 4 (0.25 pmol/well) on PC-3 cells as percent of cell-associated activity, determined via internalization studies. Incubation at 37 °C for 1 h (DMEM/F-12 + 5% BSA (*v*/*v*), 1.5 × 10^5^ cells/mL/well). Data corrected for non-specific binding (10^−3^ M [^nat^Lu]Lu-RM2); Table S2: Tumor/background ratios of ^99m^Tc-labeled MJ9 analogues for selected organs of PC-3 tumor-bearing CB17-SCID mice at 1 h p.i. (*n* = 3).
