# Supplementary material for: Optimization of the Pharmacokinetic Profile of [99mTc]Tc-N4-Bombesin Derivatives by Modification of the Pharmacophoric Gln-Trp Sequence
Source: Pharmaceuticals (Basel). 2022 Sep 10;15(9):1133. doi: 10.3390/ph15091133 (PMC9500665; doi:10.3390/ph15091133)
Supplement: Supplementary file 1 [file pharmaceuticals-15-01133-s001.zip › pharmaceuticals-1865610-supplementary.pdf]

Optimization of the Pharmacokinetic Profile of [^{99m}Tc]Tc-N$_4$-Bombesin Derivatives by Modification of the Pharmacophoric Gln-Trp Sequence

# - Supplemental Material -

Thomas Günther[1], Matthias Konrad[1], León Stopper[1], Jan-Philip Kunert[1], Sebastian Fischer[1], Roswitha Beck[1], Angela Casini[1,2], and Hans-Jürgen Wester[1]

[1]Chair of Pharmaceutical Radiochemistry, Technical University of Munich, Garching, Germany

[2]Chair of Medicinal and Bioinorganic Chemistry, Technical University of Munich, Garching, Germany

Department of Chemistry, Technical University of Munich, Garching, Germany

Corresponding author:

Thomas Günther

Phone: +49.89.289.12203

Technical University of Munich,

Chair of Pharmaceutical Radiochemistry,

Walther-Meissner-Str. 3

85748 Garching

GERMANY

Fax: +49.89.289.12204

E-Mail: thomas.guenther@tum.de

ORCID: https://orcid.org/0000-0002-7412-0297

General Information

The 9-fluorenylmethoxycarbonyl-protected (Fmoc-protected) and all other protected amino acid analogues were purchased from *Bachem Inc.* (Bubendorf, Switzerland), *Merck KGaA* (Darmstadt, Germany) or *Iris Biotech GmbH* (Marktredwitz, Germany). The *H*-Rink amide ChemMatrix® resin (35-100 mesh particle size, 0.4-0.6 mmol/g loading) was purchased from *Merck KGaA* (Darmstadt, Germany).

All necessary solvents and other organic reagents were purchased from either *Alfa Aesar™* (Karlsruhe, Germany), *Merck KGaA* (Darmstadt, Germany) or *VWR International GmbH* (Bruchsal, Germany). Solid-phase synthesis of the peptides was carried out by manual operation using a Scilogex MX-RL-E Analog Rotisserie Tube Rotator (*Scilogex®*, Rocky Hill, CT, USA). H_2O was used after purification by a Barnstead MicroPure system (*Thermo Fisher Scientific Inc.*, Waltham, MA, USA).

Analytical and preparative reversed-phase high performance liquid chromatography (RP-HPLC) were performed using Shimadzu gradient systems (*Shimadzu Deutschland GmbH*, Neufahrn, Germany), each equipped with a SPD-20A UV/Vis detector (220 nm, 254 nm). Different gradients of MeCN (0.1% TFA, 2 or 5% H_2O for analytical or preparative application, respectively) in H_2O (0.1% TFA) were used as eluents for all RP-HPLC operations.

For analytical measurements, a MultoKrom 100-5 C18 (150 mm x 4.6 mm) column (*CS Chromatographie Service GmbH*, Langerwehe, Germany) was used at a flow rate of 1 mL/min. Both, specific gradients and the corresponding retention times t_R as well as the capacity factor *K'* are cited in the text.

Preparative RP-HPLC purification was performed using a MultoKrom 100-5 C18 (250 mm x 20 mm) column (*CS Chromatographie GmbH*, Langerwehe, Germany) at a constant flow rate of 10 mL/min.

Reversed-phase high performance flash chromatography (*RP*-HPFC) was carried out on an SP HPFC system with SNAP cartridges (KP-C18-HS, 12 g) from *Biotage* (Charlottesville, VA, USA) applying H_2O (+0.1% TFA (*v*/*v*)) and MeCN (+0.1% TFA (*v*/*v*)) as eluents.

Lyophilization was accomplished using an Alpha 1-2 LDplus lyophilizer (*Martin Christ Gefriertrocknungsanlagen GmbH*, Osterode am Harz, Deutschland) combined with a RZ-2 vacuum pump (*Vacuubrand GmbH & Co KG*, Olching, Germany).

At the day of experiment, $[^{99m}Tc][TcO_4]^-$ was freshly eluted from a $^{99}Mo/^{99m}Tc$ generator using isotonic saline solution. The generator (Ultra-Technekow FM 2, 15 - 43.00 GBq, *Curium*, Petten, Netherlands) was provided by the *Klinikum rechts der Isar* (TU Munich, Germany). Radioactivity was detected through connection of the outlet of the UV photometer to an AceMate 925-Scint NaI(Tl) well-type scintillation counter from *EG&G Ortec*® (Oak Ridge, TN, USA). Radio thin layer chromatography (TLC) was performed on iTLC-SG stripes (Agilent Technologies, Waldbronn, Germany) using butanone or NH_4OAc (1 M in H_2O) with DMF (1/1 (*v*/*v*)) as mobile phase for quantification of free $[^{99m}Tc][TcO_4]^-$ or colloidal technetium-99m, respectively. Radio TLC stripes were analyzed using a Scan-RAM™ scanner with Laura™ software (*LabLogic Systems Ltd.*, Broomhill, Sheffield, UK).

Analytical and preparative radio RP-HPLC was performed using a MultoKrom 100-5 C18 (5 µm, 125 × 4.6 mm) column (*CS Chromatographie GmbH*, Langerwehe, Germany). A HERM LB 500 NaI scintillation detector (*Berthold Technologies*, Bad Wildbad, Germany) was connected to the outlet of the UV photometer for the detection of radioactivity.

Radioactive samples were measured by a WIZARD$^{2®}$ 2480 Automatic γ-Counter (*Perkin Elmer Inc.*, Waltham, MA, USA) and determination of IC_{50} values was carried out using GraphPad Prism 6 (*GraphPad Software Inc.*, San Diego, CA, USA).

For *in vitro* and *in vivo* studies, the used nutrition mixture Dulbecco's modified eagle's medium/Ham's F-12 (DMEM/F-12, *v*/*v* = 1/1, with stable glutamine), fetal bovine serum (FBS Superior), phosphate buffered saline (PBS Dulbecco, without Ca^{2+}/Mg^{2+}), trypsin/EDTA (0.05%/0.02% in PBS without Ca^{2+}/Mg^{2+}) solution as well as Hank's balanced salt solution (HBSS, with 0.35 g/L $NaHCO_3$ and Ca^{2+}/Mg^{2+}) were obtained from *Biochrom GmbH* (Berlin, Germany). Solutions of purified products were prepared using Tracepur® H_2O (*Merck KGaA*, Darmstadt, Germany). Bovine serum albumin (BSA) was purchased from *Merck KGaA* (Darmstadt, Germany).

Cells were cultivated in CELLSTAR® cell culture flasks and seeded in 24-well plates (*Greiner Bio-One GmbH*, Kremsmünster, Austria) after being counted with a Neubauer hemocytometer (*Paul Marienfeld*, Lauda-Königshofen, Germany) using Trypan Blue (0.4% in 0.81% NaCl and 0.06% potassium phosphate) solution (*Sigma-Aldrich GmbH*, Munich, Germany). Cells were handled inside a MSC Advantage laminar flow cabinet and maintained in a Heracell 150i incubator (*Thermo Fisher Scientific Inc.*, Waltham, MA, USA) at 37 °C in a humidified 5% CO_2 atmosphere.

General Procedures (GP) and Execution Protocols

On-Resin Peptide Bond Formation (GP1). The respective side-chain protected Fmoc-AA-OH (1.5 eq.) is dissolved in NMP and pre-activated by adding TBTU (1.5 eq.), HOAt (1.5 eq.) and DIPEA (4.5 eq.). After activation for 10 min, the solution is added to resin-bound free amine peptide and shaken for 1.5 h at room temperature. Subsequently, the resin is washed with

NMP (6 × 20 mL/g resin) and after Fmoc deprotection (GP2), the next amino acid is coupled analogously.

On-Resin Fmoc Deprotection (GP2). The resin-bound Fmoc-protected peptide is treated with 20% piperidine in NMP (*v*/*v*) for 5 min and subsequently for 15 min. Afterwards, the resin is washed with NMP (6 × 20 mL/g resin).

Conjugation of Chelator (GP3). The protected tetraamine chelator $N_4(Boc)_4$ (1.5 eq.) is dissolved in NMP and pre-activated by adding TBTU (1.5 eq.), HOAt (1.5 eq.) and DIPEA (4.5 eq.). After activation for 10 min, the solution is added to resin-bound *N*-terminal deprotected peptide (1.0 eq.) and shaken for 3 h at room temperature. Subsequently, the resin is washed with NMP (3 × 20 mL/g resin) and DCM (3 × 20 mL/g resin).

Peptide Cleavage from the Resin with Simultaneous Deprotection of Acid Labile Protecting Groups (GP4). The fully protected resin-bound peptide is washed with DCM, afterwards a mixture of TFA/TIPS/DCM (*v*/*v*/*v*; 95/2.5/2.5) is added and the resin is shaken for 45 min. The solution is filtered off and the resin is treated in the same way for another 45 min. Both filtrates are combined and concentrated under a stream of nitrogen. After dissolving the residue in MeOH, precipitation in diethyl ether and centriguation, the liquid is decanted and the remaining solid is dried.

Radiolabeling

^{125}I-Labeling. Briefly, 0.2 mg of Tyr^{6}-MJ9 were dissolved in 20 µL Tracepur® H_2O and 280 µL TRIS buffer (25 mM TRIS · HCl, 0.4 M NaCl, pH = 7.9). After the solution was transferred to a vial containing 150 µg surface-bound Iodo-Gen® (1,3,4,6-Tetrachloro-3*α*,6*α*-diphenylglycouril, *Merck KGaA*, Darmstadt, Germany), 5.0 µL (16 MBq) ^{125}I-NaI (74 TBq/mmol, 3.1 GBq/mL, 40 mM NaOH, *Hartmann Analytic*, Braunschweig, Germany) were added. The reaction

solution was incubated for 15 min at room temperature and purified by RP-HPLC. Immediately after purification, sodium ascorbate (0.1 M in Tracepur® H_2O, 10 vol-%) was added to prevent radiolysis.

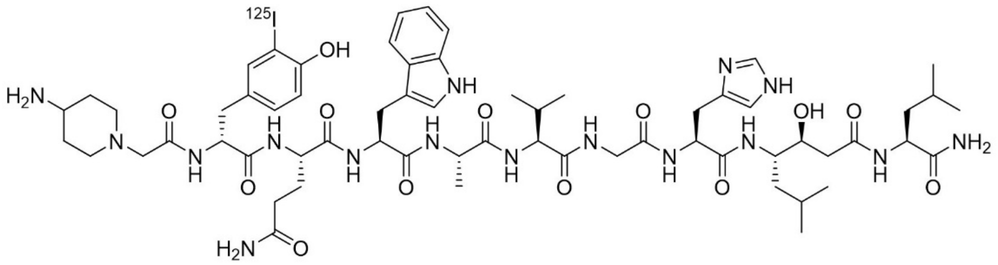

Figure S1. Structural formula of the radiolabeled counterpart (3-[^{125}I]I-tyr^{6}-MJ9) of the non-radioactive competitor (3-I-tyr^{6}-MJ9) in IC_{50} studies.

3-[^{125}I]I-tyr^{6}-MJ9. RP-HPLC (20→35% MeCN in 20 min). t_R = 18.5 min, K' = 12.21.

3-I-tyr^{6}-MJ9. RP-HPLC (20→35% MeCN in 20 min). t_R = 18.4 min, K' = 12.14.

Calculated monoisotopic mass ($C_{78}H_{118}N_{20}O_{19}$): 1394.6, found: m/z = 698.3 $[M+2H]^{2+}$, 1394.9 $[M+H]^{+}$.

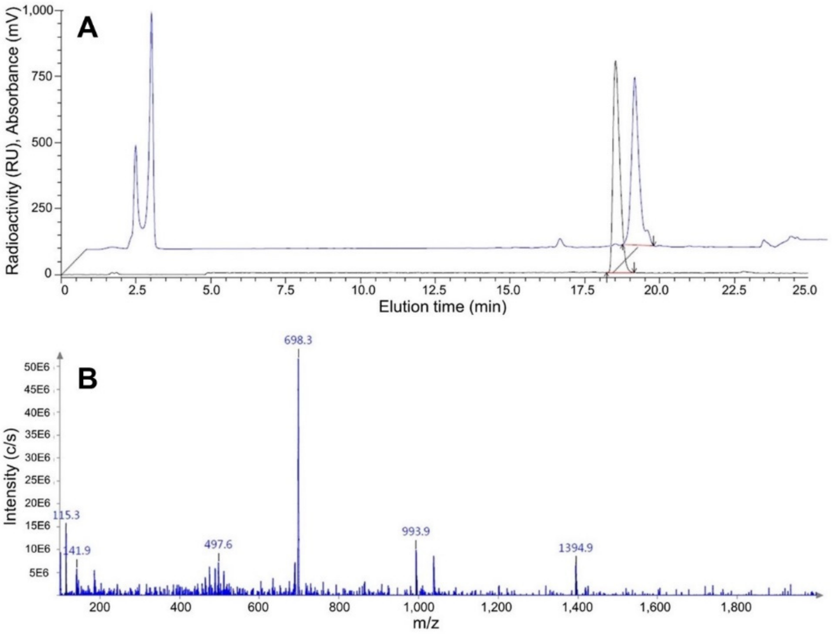

Figure S2. a) Confirmation of peptide integrity for 3-^{125}I-tyr^{6}-MJ9 (black), as analyzed by analytical radio RP-HPLC via co-injection of the non-radioactive ligand (blue). b) Mass spectrum of 3-I-tyr^{6}-MJ9.

Characterization of N$_4$-conjugated MJ9 analogues

All MJ9 derivatives were synthesized by standard Fmoc-based SPPS (**GP1-4**) using a *H*-Rink amide ChemMatrix® resin (35-100 mesh particle size, 0.4-0.6 mmol/g loading, *Merck KGaA*, Darmstadt, Germany). After finishing the peptide sequence with slightly modifications within the MJ9 sequence, the tetraamine chelator N$_4$ was coupled on-resin (**GP3**). Thereafter, the peptide was cleaved and deprotected (**GP4**) and subsequently purified by RP-HPLC.

N$_4$-asp-MJ9 (N$_4$-asp-Pip-phe-Gln-Trp-Ala-Val-Gly-His-Sta-Leu-NH$_2$)

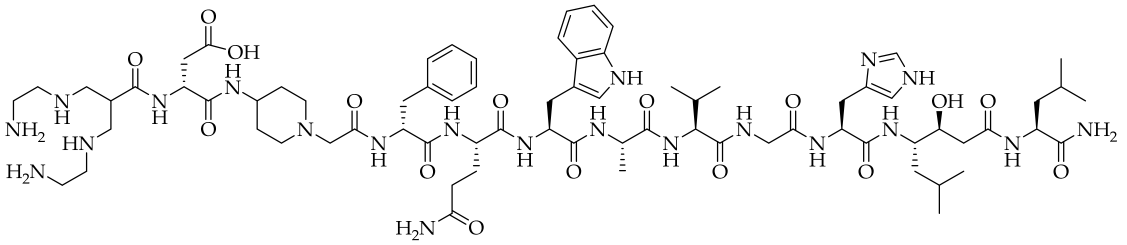

Figure S3. Structural formula of N$_4$-asp-MJ9.

Unlabeled. RP-HPLC (20→35% MeCN in 15 min): t_R = 11.8 min, *K'* = 7.43.

Calculated monoisotopic mass ($C_{74}H_{115}N_{21}O_{16}$): 1553.9, found: m/z = 519.9 $[M+3H]^{3+}$, 779.3 $[M+2H]^{2+}$.

^{99m}Tc-Labeled. RP-HPLC (20→35% MeCN in 15 min): t_R = 13.0 min, *K'* = 8.29.

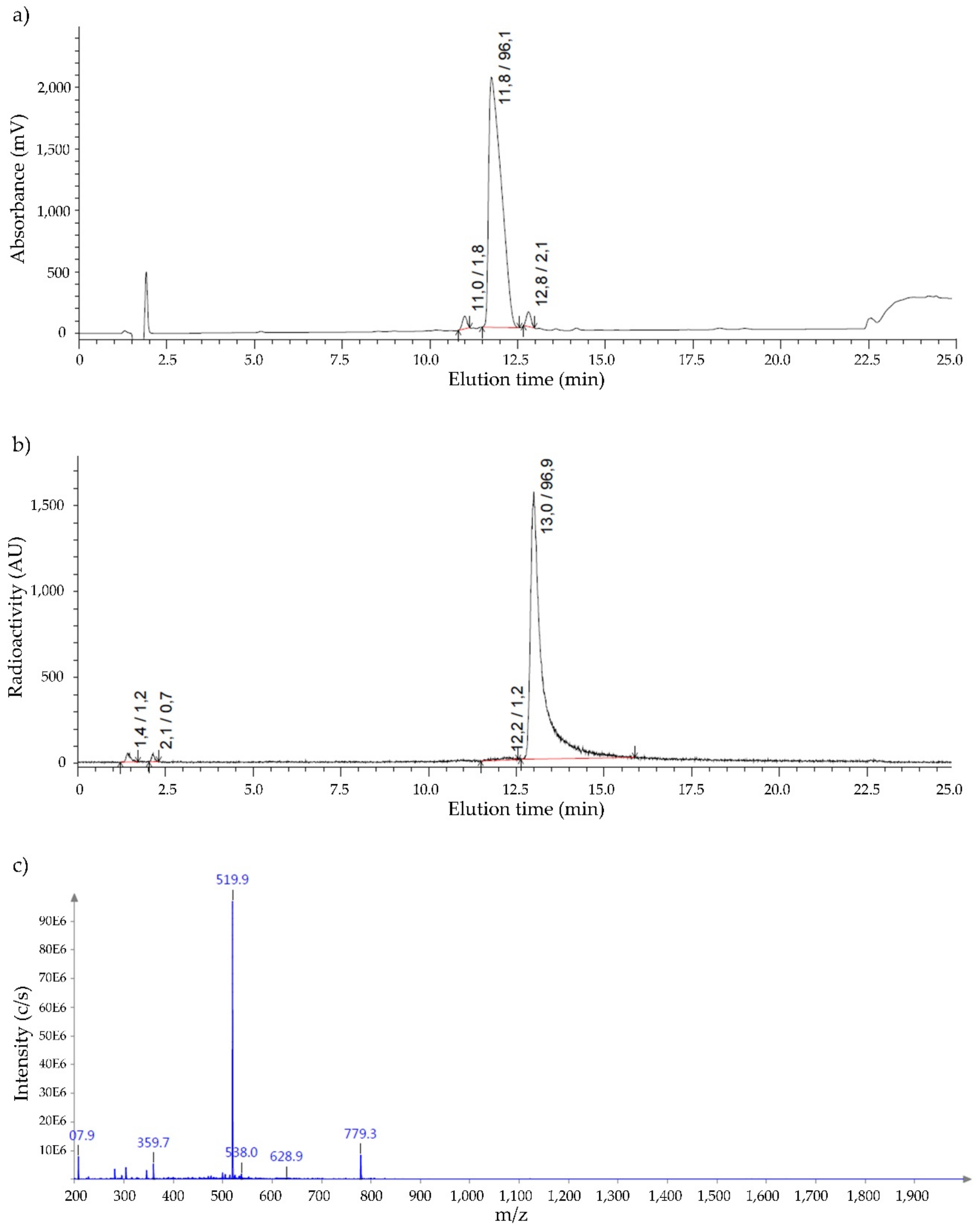

Figure S4. a) Confirmation of peptide identity and integrity for N4-asp-MJ9, as analyzed by analytical RP-HPLC. b) Confirmation of peptide identity and integrity for [^{99m}Tc]Tc-N4-asp-MJ9, as analyzed by analytical radio RP-HPLC. c) Mass spectrum of N4-asp-MJ9.

N4-asp-[Bta^{8}]MJ9 (N4-asp-Pip-phe-Gln-Bta-Ala-Val-Gly-His-Sta-Leu-NH_2)

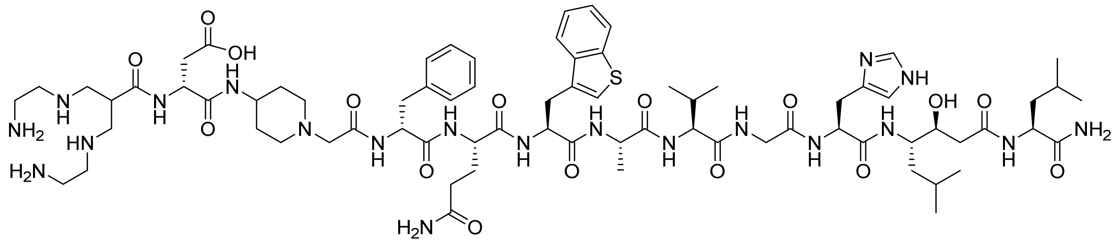

Figure S5. Structural formula of N4-asp-[Bta8]MJ9.

Unlabeled. RP-HPLC (20→35% MeCN in 15 min): t_R = 13.9 min, K' = 8.93.

Calculated monoisotopic mass ($C_{74}H_{114}N_{20}O_{16}S$): 1570.8, found: m/z = 525.6 $[M+3H]^{3+}$, 787.8 $[M+2H]^{2+}$.

^{99m}Tc-Labeled. RP-HPLC (20→35% MeCN in 15 min): t_R = 15.4 min, K' = 10.00.

a)

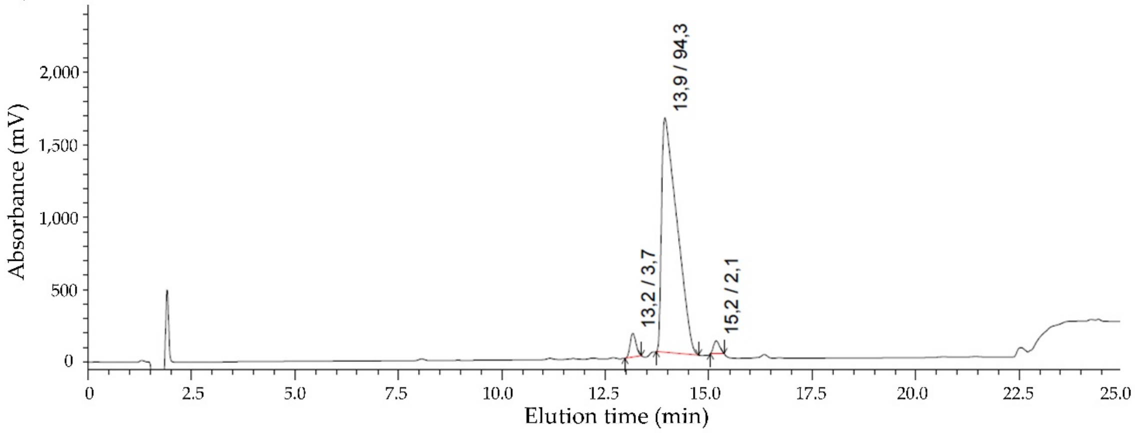

b)

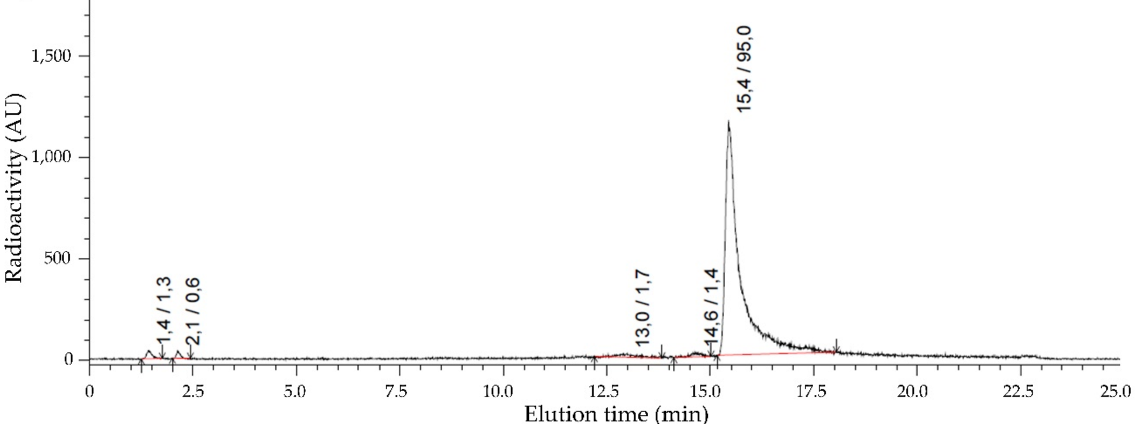

c)

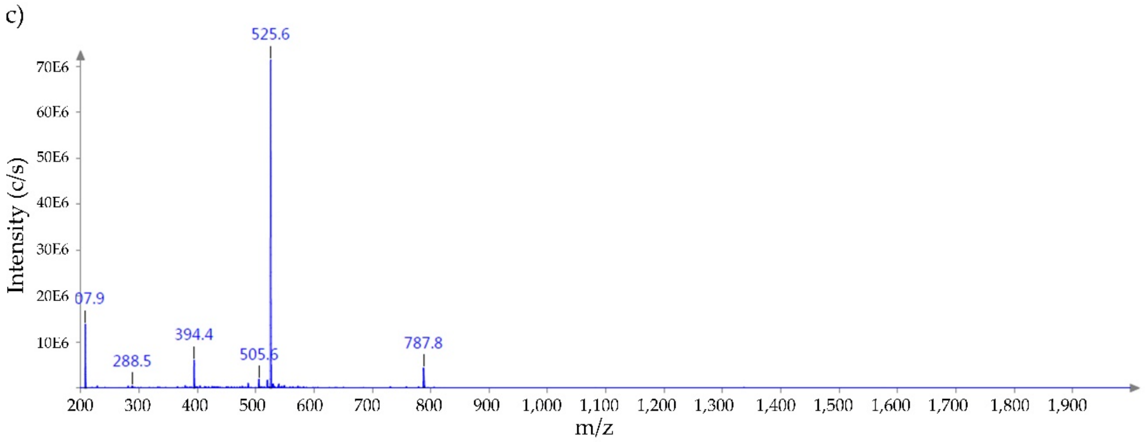

Figure S6. a) Confirmation of peptide identity and integrity for N_4-asp-[Bta^8]MJ9, as analyzed by analytical RP-HPLC. b) Confirmation of peptide identity and integrity for [^{99m}Tc]Tc-N_4-asp-[Bta^8]MJ9, as analyzed by analytical radio RP-HPLC. c) Mass spectrum of N_4-asp-[Bta^8]MJ9.

N_4-[Hse^7]MJ9 (N_4-Pip-phe-Hse-Trp-Ala-Val-Gly-His-Sta-Leu-NH_2)

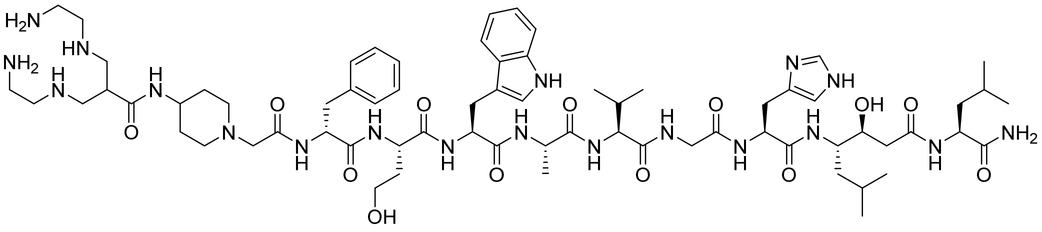

Figure S7. Structural formula of N_4-[Hse^7]MJ9.

Unlabeled. RP-HPLC (20→35% MeCN in 15 min): t_R = 12.7 min, K' = 8.07.

Calculated monoisotopic mass ($C_{69}H_{109}N_{19}O_{13}$): 1411.9, found: m/z = 472.4 $[M+3H]^{3+}$, 708.2 $[M+2H]^{2+}$.

^{99m}Tc-Labeled. RP-HPLC (20→35% MeCN in 15 min): t_R = 13.9 min, K' = 8.93.

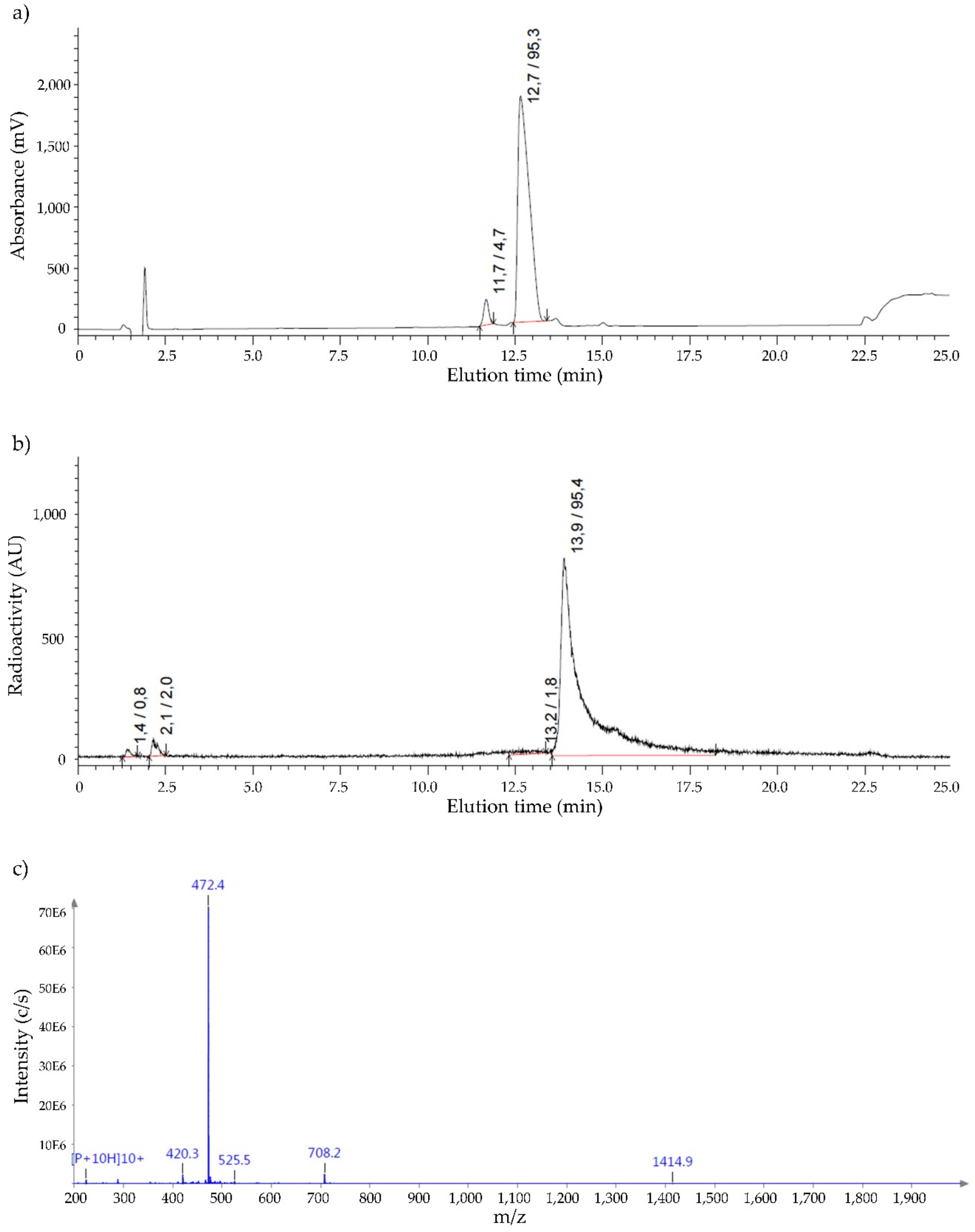

Figure S8. a) Confirmation of peptide identity and integrity for N_4-[Hse^7]MJ9, as analyzed by analytical RP-HPLC. b) Confirmation of peptide identity and integrity for [^{99m}Tc]Tc-N_4-[Hse^7]MJ9, as analyzed by analytical radio RP-HPLC. c) Mass spectrum of N_4-[Hse^7]MJ9.

N_4-[α-Me-Trp^8]MJ9 (N_4-Pip-phe-Gln-α-Me-Trp-Ala-Val-Gly-His-Sta-Leu-NH_2)

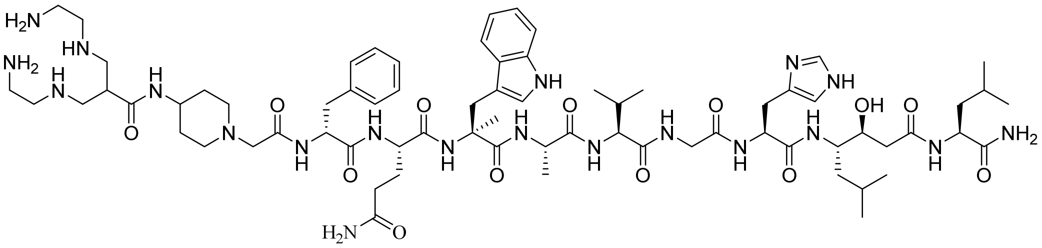

Figure S9. Structural formula of N4-[α-Me-Trp[8]]MJ9.

Unlabeled. RP-HPLC (20→35% MeCN in 15 min): t_R = 13.7 min, K' = 8.79.

Calculated monoisotopic mass ($C_{71}H_{112}N_{20}O_{13}$): 1452.9, found: m/z = 486.1 $[M+3H]^{3+}$, 728.6 $[M+2H]^{2+}$. Structural formula of N4-[α-Me-Trp[8]]MJ9

^{99m}Tc-Labeled. RP-HPLC (20→35% MeCN in 15 min): t_R = 14.6 min, K' = 9.43.

a)

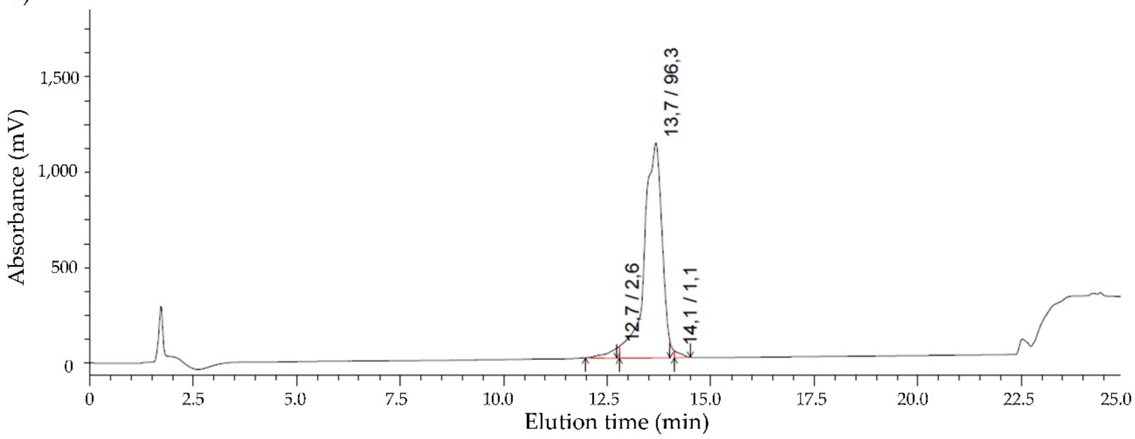

b)

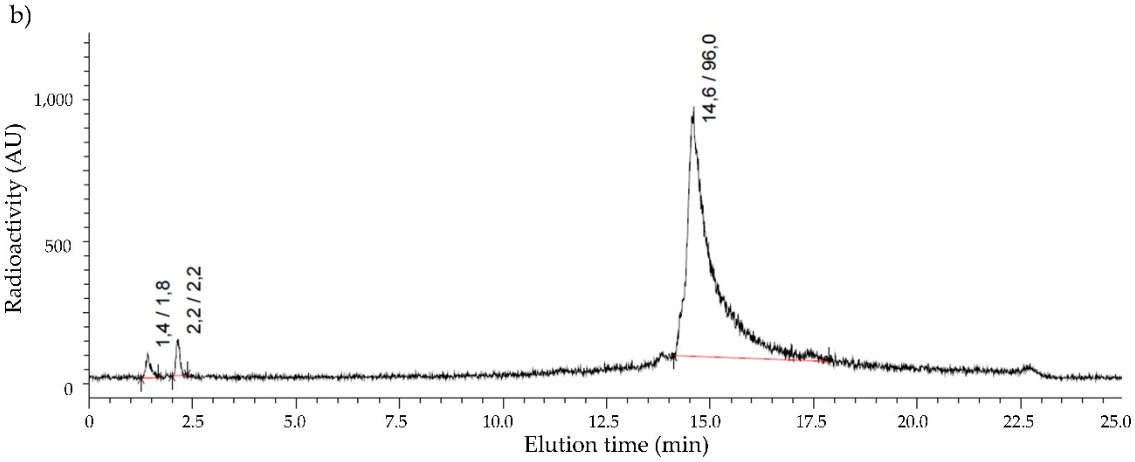

c)

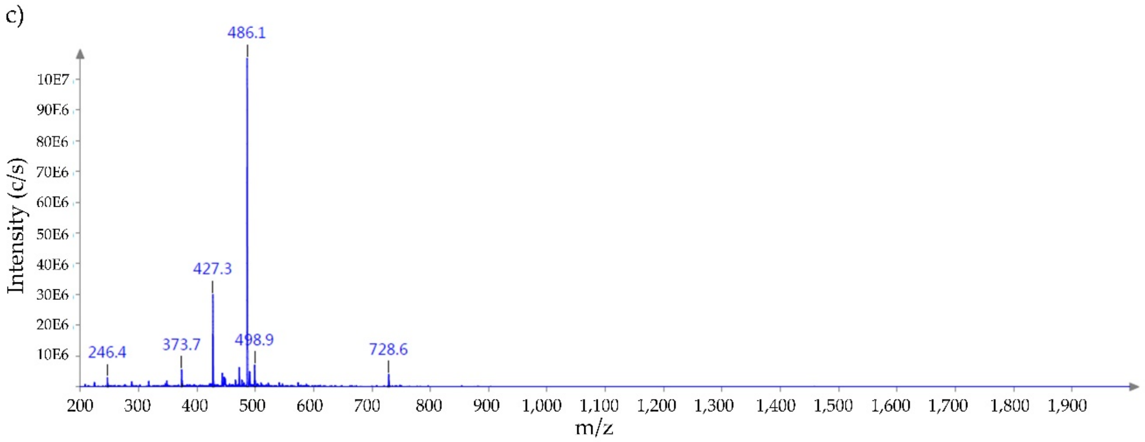

Figure S10. a) Confirmation of peptide identity and integrity for N4-[α-Me-Trp^8]MJ9, as analyzed by analytical RP-HPLC. b) Confirmation of peptide identity and integrity for [^{99m}Tc]Tc-N4-[α-Me-Trp^8]MJ9, as analyzed by analytical radio RP-HPLC. c) Mass spectrum of N4-[α-Me-Trp^8]MJ9.

Demobesin 4 (N4-Pro-Gln-Arg-Tyr-Gly-Asn-phe-Gln-Trp-Ala-Val-Gly-His-Leu-Nle-NH2)

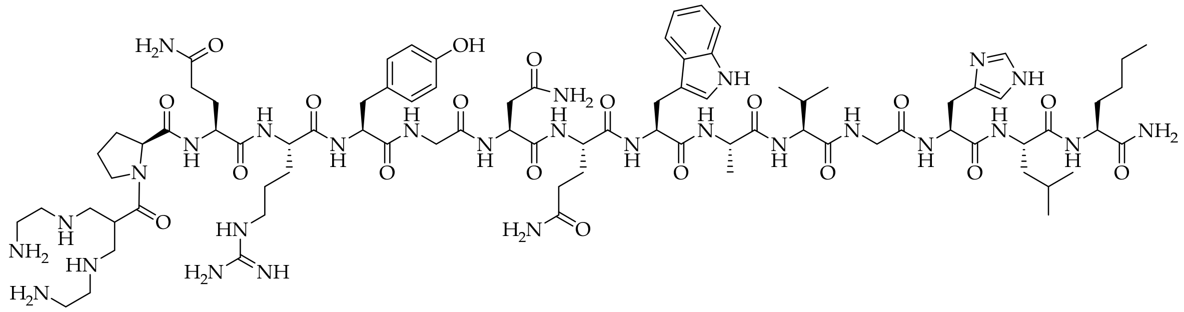

Figure S11. Structural formula of the external reference Demobesin 4.

Unlabeled. RP-HPLC (20→35% MeCN in 15 min): t_R = 13.7 min, K' = 8.79.

Calculated monoisotopic mass ($C_{83}H_{130}N_{28}O_{19}$): 1823.0, found: m/z = 457.5 $[M+4H]^{4+}$, 609.7 $[M+3H]^{3+}$.

^{99m}Tc-Labeled. RP-HPLC (20→35% MeCN in 15 min): t_R = 10.9 min, K' = 6.79.

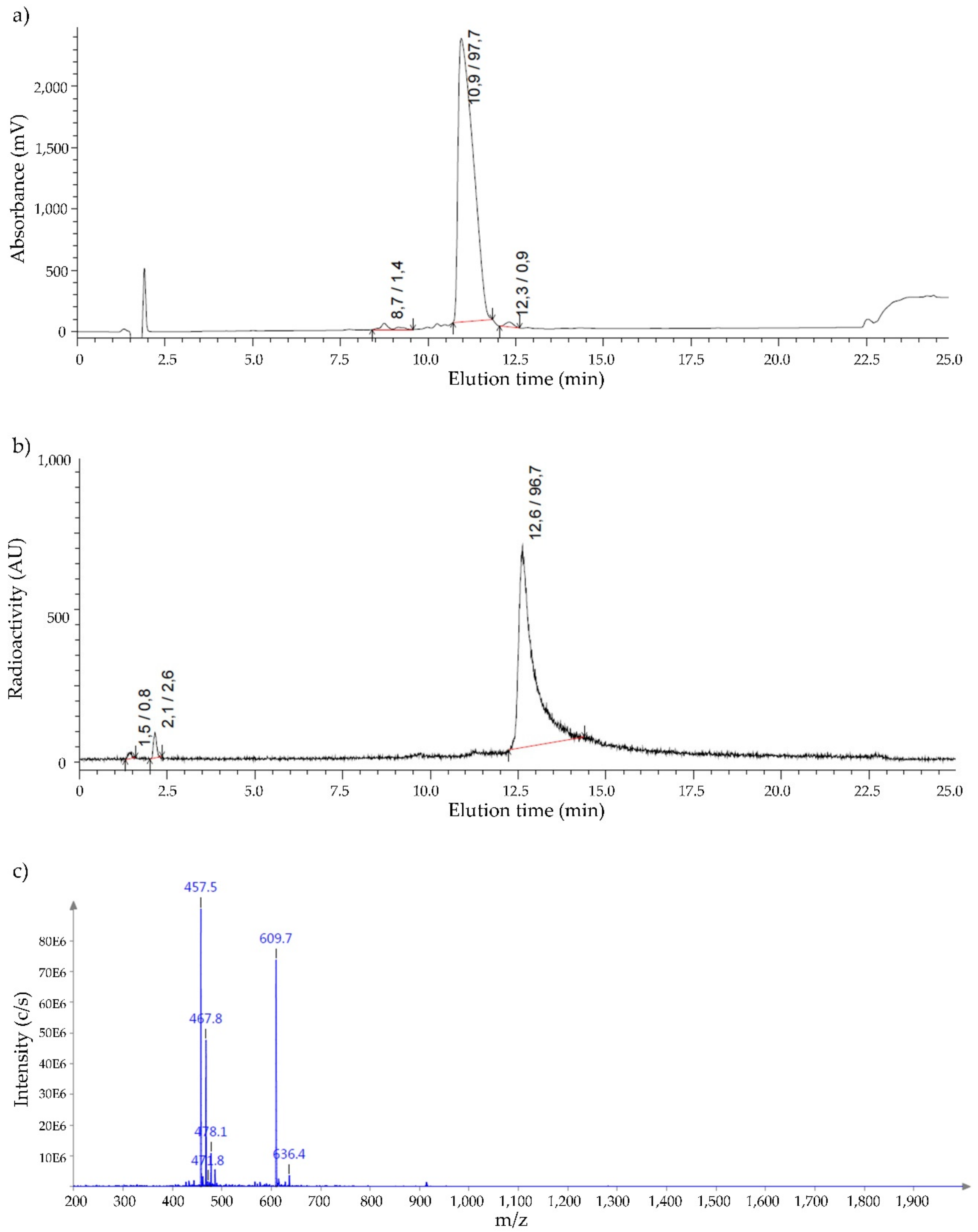

Figure S12. a) Confirmation of peptide identity and integrity for Demobesin 4, as analyzed by analytical RP-HPLC. b) Confirmation of peptide identity and integrity for [^{99m}Tc]Tc-Demobesin 4, as analyzed by analytical radio RP-HPLC. c) Mass spectrum of Demobesin 4.

***In Vitro* Experiments**

Cell Culture. $GRPR^+$ PC-3 cells (*Merck KGaA*, Darmstadt, Germany) were cultivated in Dulbecco's modified eagle's medium/Ham's F-12 (DMEM/F-12, *v/v* = 1/1, with stable glutamine, *Bio&SELL GmbH*, Feucht, Germany) supplemented with fetal bovine serum (10%, FBS Superior, *Bio&SELL GmbH, Feucht, Germany*) at 37 °C in a humidified 5% CO_2 atmosphere. A mixture of trypsin and ethylenediaminetetraacetic acid (0.05%, 0.02%) in PBS (*Biochrom GmbH*, Berlin, Germany) was used in order to harvest cells. Cells were counted with a Neubauer hemocytometer (*Paul Marienfeld*, Lauda-Königshofen, Germany).

Determination of inverse IC_{50}. For determination of GRPR affinity on PC-3 cells ($IC_{50,\mathrm{inverse}}$), cells were harvested and seeded in 24-well plates (1.5×10^5 cells in 1 mL/well) 24 ± 2 h before the experiment.

After removal of the culture medium, the cells were washed once with 500 µL of HBSS (Hank's balanced salt solution, *Biochrom GmbH*, Berlin, Germany, with addition of 1% bovine serum albumin (BSA, *v/v*)) and left in 200 µL HBSS (1% BSA, *v/v*) for 9 min at room temperature for equilibration. Next, 25 µL per well of solutions, containing either HBSS (1% BSA, *v/v*) as control or the non-radioactive competitor (3-I-tyr^6-MJ9) in increasing concentration (10^{-10}-10^{-4} M in HBSS (1% BSA, *v/v*)), were added with subsequent addition of 25 µL of the respective ^{99m}Tc-labeled compound (0.2 nM/well) in HBSS (1% BSA, *v/v*).

All experiments were performed in triplicate for each concentration. After 2 h incubation at room temperature, the experiment was terminated by removal of the medium and consecutive rinsing with 300 µL of HBSS (1% BSA, *v/v*). The media of both steps were combined in one fraction and represent the amount of free ^{99m}Tc-labeled compound. Afterwards, the cells were lysed with 300 µL of 1 M NaOH for at least 15 min and united with the 300 µL NaOH of the following washing step. Quantification of bound and free ^{99m}Tc-

labeled compound was accomplished in a γ-counter. IC_{50} determination for each conjugate was repeated twice.

Receptor-mediated Internalization. For internalization studies, PC-3 cells were harvested and seeded in poly-L-lysine coated 24-well plates (1.5×10^5 cells/well, 1 mL, *Greiner Bio-One*, Kremsmünster, Austria) 24 ± 2 h before the experiment. Subsequent to the removal of the culture medium, the cells were washed once with 500 µL DMEM/F-12 (5% BSA, *v/v*) and left to equilibrate at 37 °C for at least 15 min in 200 µL DMEM/F-12 (5% BSA, *v/v*). Each well was treated with either 25 µL of DMEM/F-12 (5% BSA, *v/v*) or 25 µL ^{nat}Lu-RM2 (10^{-3} M in Tracepur® H_2O) for blockade. Next, 25 µL of the ^{99m}Tc-labeled MJ9 analogue (10 nM) was added and the cells were incubated at 37 °C for 60 min.

The experiment was terminated by placing the 24-well plate on ice for 1 min and consecutive removal of the medium. Each well was rinsed with 300 µL ice-cold PBS and the fractions from these first two steps were combined, representing the amount of free ^{99m}Tc-labeled MJ9 analogue. Removal of surface bound activity was accomplished by incubation of the cells with 300 µL of ice-cold Acid Wash solution (0.02 M NaOAc, pH = 5.0) for 10 min at room temperature and rinsed again with 300 µL of ice-cold PBS. The internalized activity was determined by incubation of the cells in 300 µL NaOH (1 M) and the combination with the fraction of a subsequent washing step with 300 µL NaOH (1 M).

Each experiment (control and blockade) was performed sixfold. Free, surface bound and internalized activity was quantified in a γ-counter. Data was corrected for non-specific internalization.

Supplemental Data

Table S1. Preclinical data of [^{99m}Tc]Tc-N$_4$-asp-MJ9, [^{99m}Tc]Tc-N$_4$-asp-[Bta^8]MJ9, [^{99m}Tc]Tc-N$_4$-[Hse^7]MJ9, [^{99m}Tc]Tc-N$_4$-[α-Me-Trp^8]MJ9 and [^{99m}Tc]Tc-Demobesin-4. Affinity data of the respective ^{99m}Tc-labeled ligand (8.0 nM/well) was determined on PC-3 cells (1.5 × 10^5 cells/well) and 3-I-tyr^6-MJ9 (10^{-10}-10^{-4} M) as non-radioactive competitor (2 h, room temperature, HBSS + 1% BSA, *v*/*v*). Lipophilicity was examined as distribution coefficient in a mixture of n-octanol/PBS (*v*/*v* = 1/1) at pH 7.4 (log$D_{7.4}$). Cell membrane-bound fraction of [^{99m}Tc]Tc-N$_4$-asp-MJ9, [^{99m}Tc]Tc-N$_4$-asp-[Bta^8]MJ9, [^{99m}Tc]Tc-N$_4$-[Hse^7]MJ9, [^{99m}Tc]Tc-N$_4$-[α-Me-Trp^8]MJ9 and [^{99m}Tc]Tc-Demobesin 4 (0.25 pmol/well) on PC-3 cells as percent of cell-associated activity, determined via internalization studies. Incubation at 37°C for 1 h (DMEM/F-12 + 5% BSA (*v*/*v*), 1.5 × 10^5 cells/mL/well). Data corrected for non-specific binding (10^{-3} M [^{nat}Lu]Lu-RM2).

GRPR-targeted compound	$IC_{50,inverse}$ (nM)	log$D_{7.4}$	Membrane-bound fraction [% of cell-associated activity]
	n = 3	n = 6	n = 6
**[^{99m}Tc]Tc-N$_4$-asp-MJ9**	2.5 ± 0.8	−1.86 ± 0.03	81.5 ± 0.4
**[^{99m}Tc]Tc-N$_4$-asp-[Bta^8]MJ9**	6.5 ± 1.1	−1.33 ± 0.06	79.5 ± 0.6
**[^{99m}Tc]Tc-N$_4$-[Hse^7]MJ9**	2.6 ± 0.1	−1.26 ± 0.02	81.0 ± 0.7
**[^{99m}Tc]Tc-N$_4$-[α-Me-Trp^8]MJ9**	5.5 ± 0.5	−1.14 ± 0.04	82.5 ± 0.7
**[^{99m}Tc]Tc-Demobesin 4**	2.7 ± 0.9	−2.10 ± 0.02	29.3 ± 2.1

Table S2. Tumor/background ratios of ^{99m}Tc-labeled MJ9 analogues for selected organs of PC-3 tumor-bearing CB17-SCID mice at 1 h p.i. (n = 3).

Organ	[^{99m}Tc]Tc-N$_4$-asp-MJ9	[^{99m}Tc]Tc-N$_4$-asp-[Bta^8]MJ9	[^{99m}Tc]Tc-N$_4$-[Hse^7]MJ9	[^{99m}Tc]Tc-N$_4$-[α-Me-Trp^8]MJ9
**Blood**	18.4 ± 8.4	10.6 ± 4.6	21.5 ± 6.0	7.6 ± 0.3
**Heart**	46.7 ± 19.3	26.8 ± 9.5	42.1 ± 8.5	17.6 ± 0.4
**Lung**	19.0 ± 6.9	12.6 ± 4.1	18.9 ± 5.6	6.5 ± 0.0
**Liver**	26.7 ± 7.6	11.4 ± 2.8	5.2 ± 0.7	4.5 ± 0.0
**Spleen**	42.6 ± 14.5	22.6 ± 4.4	25.7 ± 6.0	10.0 ± 0.7
**Pancreas**	0.7 ± 0.1	2.0 ± 0.4	1.7 ± 0.6	0.2 ± 0.0
**Stomach**	6.7 ± 1.20	8.9 ± 1.0	7.5 ± 0.9	4.3 ± 0.0
**Intestine**	6.2 ± 1.6	11.9 ± 4.4	5.9 ± 1.1	2.7 ± 0.3
**Kidney**	4.0 ± 0.9	2.9 ± 0.6	3.9 ± 0.7	2.1 ± 0.2
**Adrenal**	44.5 ± 63.6	9.9 ± 5.5	6.5 ± 2.7	10.1 ± 3.8
**Muscle**	85.0 ± 33.7	35.4 ± 18.6	98.2 ± 18.2	28.0 ± 10.8
**Bone**	45.2 ± 15.1	38.1 ± 21.3	73.4 ± 20.9	15.5 ± 0.4
